# Sex‐stratified genome‐wide meta‐analysis identifies novel loci for cognitive decline in older adults

**DOI:** 10.1002/alz.14461

**Published:** 2025-03-05

**Authors:** Vibha Acharya, Kang‐Hsien Fan, Beth E. Snitz, Mary Ganguli, Steven T. DeKosky, Oscar L. Lopez, Eleanor Feingold, M. Ilyas Kamboh

**Affiliations:** ^1^ Department of Human Genetics University of Pittsburgh School of Public Health Pittsburgh Pennsylvania USA; ^2^ Department of Neurology School of Medicine University of Pittsburgh Pittsburgh Pennsylvania USA; ^3^ Department of Psychiatry School of Medicine University of Pittsburgh Pittsburgh Pennsylvania USA; ^4^ Department of Epidemiology University of Pittsburgh School of Public Health Pittsburgh Pennsylvania USA; ^5^ McKnight Brain Institute and Department of Neurology College of Medicine University of Florida Gainesville Florida USA

**Keywords:** cognitive decline, females, males, *NDUFA12*, neurocognitive domains, sex‐stratified genome‐wide association

## Abstract

**INTRODUCTION:**

Many complex traits and diseases show sex‐specific biases in clinical presentation and prevalence.

**METHODS:**

To understand sex‐specific genetic architecture of cognitive decline across five cognitive domains (attention, memory, executive function, language, and visuospatial function) and global cognitive function, we performed sex‐stratified genome‐wide meta‐analysis in 3021 older adults aged ≥ 65 years (female = 1545, male = 1476) from three prospective cohorts. Gene‐based and gene‐set enrichment analyses were conducted for each cognitive trait.

**RESULTS:**

We identified a novel genome‐wide significant (GWS) intergenic locus for decline of memory in males near *RPS23P3* on chromosome 4 (rs6851574: minor allele frequency [MAF] = 0.39, *P*_male_ = 4.10E‐08, *β*_male_ = 0.19; *P*_interaction_ = 3.76E‐04). We also identified a subthreshold GWS locus for decline of executive function on chromosome 12 in females near the *NDUFA12* gene, involved in oxidative phosphorylation (rs11107823: MAF = 0.12, *P*_female_  = 9.35E‐08, *β*_female_ = 0.28; *P*_interaction_ = 7.42E‐06).

**DISCUSSION:**

Sex‐aware genetic studies can help in the identification of novel genetic loci and enhance sex‐specific understanding of cognitive decline.

**Highlights:**

- Our genome‐wide meta‐analysis of single variants identified two new genetic associations, one in males and one in females.
- The novel male association was observed with the decline of memory in the intergenic region near the *RPS23P3* gene on chromosome 4. This intergenic region has previously been implicated in several brain and cognition related traits, including anatomical brain aging, brain shape, and educational attainment.
- The novel female‐specific association was observed with decline in executive function on chromosome 12 near the *NDUFA12* gene, which is involved in oxidative phosphorylation.
- Sex‐stratified analyses offer sex‐specific understanding of biological pathways involved in cognitive aging.

## 1 BACKGROUND

Several complex traits and diseases, including neurocognitive disorders, exhibit sex‐specific differences in disease prevalence as well as progression, including neurocognitive disorders.^1^, ^2^, ^3^ Women comprise two thirds of Alzheimer's disease (AD) cases. Along with disproportionate prevalence, the clinical risk of AD is also different between sexes. A unit increase in AD pathology in women was found to increase AD risk by 20 times compared to a 3‐fold increased risk in men.^4^ Although the greater ratio of female AD cases to males was previously attributed to longer life span, hormones, and other environmental factors, emerging evidence suggests sex‐specific genetic differences could also contribute to the differing manifestations and prevalence of AD between the sexes.^5^, ^6^, ^7^

As with AD pathology, age‐related cognitive traits across several cognitive domains also exhibit sex‐specific effects.^8^ Men are found to outperform women on spatial tasks, while women are found to perform better than men on tasks related to verbal ability and social functioning.^9^, ^10^ A longitudinal study conducted on individuals > 64 years of age to understand sex‐based differences in cognitive aging found that men had a steeper decline of visuospatial ability and perceptuomotor abilities.^11^ Other studies have reported women to have a higher cognitive reserve, but a steeper cognitive decline in older age across cognitive domains and global cognitive function.^8^, ^12^ A recent genetic association study on cognitive empathy identified a locus specific to women, indicating that cognitive phenotypes might have a distinct sex‐specific genetic architecture.^13^ Studies on longevity, cognitive reserve, and cognitive decline have also identified genetic loci with differential effects between males and females.^14^, ^15^, ^16^ Another study found that common variations in the complement component C4 gene might induce a higher risk for men compared to women for systemic lupus erythematosus, Sjögren's syndrome, and schizophrenia.^17^ Polygenic risk scores for autism have also shown that the risk for this complex disease might be dimorphic among the sexes.^18^ Additionally, gene expression studies in males and females have shown that the change in gene expression during older age is dimorphic, with men showing downregulation of genes involved in energy production and protein synthesis, and women showing downregulation in neuronal morphogenesis genes.^19^ These findings suggest that sex‐stratified genome‐wide association studies (GWAS) might help us to understand the sex‐specific genetic contributors of cognitive phenotypes and elucidate molecular mechanisms underlying sex‐specific cognitive trajectories. The objective of our study was to understand the sex‐specific genetic architecture of cognitive decline across biological sexes.

We leveraged three prospective study cohorts to conduct meta‐analyses and assess the sex‐specific genetic association of cognitive decline across five cognitive domains and global function. We performed sex‐stratified genome‐wide association analyses using individual variations in slopes followed by gene‐based and gene‐set analyses. The sex‐stratified analyses enabled us to identify two novel associations, one in males and one in females. The identification of sex‐specific variants, genes, and biological pathways driving age‐related cognitive decline will contribute to our understanding of the genetic architecture of cognitive decline in each of the sexes and aid in precision medicine approaches.

## 2 METHODS

### 2.1 Participants and cohort description

The study included 3021 participants (1476 male and 1545 female) from three cohorts with longitudinal cognitive assessment: the Gingko Evaluation of Memory (GEM) study, the Monongahela‐Youghiogheny Healthy Aging Team (MYHAT), and the Monongahela Valley Independent Elders Survey (MoVIES). The demographic characteristics of the individuals across cohorts are described in Table 1, including the baseline and final visit Clinical Dementia Rating (CDR). At baseline all subjects had CDR of 0 or 0.5. Only 38 subjects had a final CDR of ≥ 1, including 14 in GEM and 24 in MYHAT. Briefly, GEM was a randomized, placebo‐controlled trial that was conducted from 2000 to 2008 across five different academic centers in the United States to assess the effectiveness of Ginkgo biloba on incident dementia in 3069 participants aged ≥ 75, including 2587 cognitively normal individuals and 482 individuals with mild cognitive impairment (MCI).^20^ The GEM participants were followed annually for a mean follow‐up of 6.11 (standard deviation [SD] = 0.98, range = 1–7.5 years, range of assessments/visits = 2–9) years, during which cognitive phenotypes were collected. Participants who had only a baseline visit were excluded from the current study. The incident dementia was determined by the panel of experts consisting of neurologists, neuropsychologists, psychometricians, and neuroradiologists based on a neuropsychological battery of tests, CDR rating, along with evaluation of neuro‐images (magnetic resonance imaging). Due to the relatively small sample size and to avoid false positives due to population stratification, individuals belonging to any race other than non‐Hispanic Whites (NHWs) were also excluded from the analysis. Thus, a total of 1898 (840 female and 1058 male) GEM participants were eligible for the genetic analysis.

**TABLE 1 alz14461-tbl-0001:** Demographic of participants in three cohorts.

	GEM (*n* = 1898)		MYHAT (*n* = 745)		MoVIES (*n* = 378)	
	Females	Males	Females	Males	Females	Males
Mean age (years [SD])	78.1 (3.1)	78.2 (3)	77.1 (7.4)	77.3 (7.2)	77.9(4.5)	76.5(3.4)
Education (mean [SD])	14.1(2.7)	14.6(3)	12.7(2.2)	13.5(2.8)	11.7(2.1)	12(2.5)
Attention slopes (mean [SD])	−0.010 (0.042)	−0.0128 (0.045)	−0.0116 (0.041)	−0.010 (0.034)	1.55E‐10 (0.049)	1.61E‐11 (0.049)
Executive function slopes (mean [SD])	−0.011 (0.037)	−0.014 (0.039)	−0.017 (0.072)	−0.0077 (0.062)	−1.55E‐11 (0.037)	1.82E‐19 (0.031)
Memory slopes (mean [SD])	−0.017 (0.062)	−0.0139 (0.059)	−0.00317 (0.090)	0.00407 (0.09)	3.9E‐12 (0.066)	−8.06E‐11 (0.069)
Language slopes (mean [SD])	−0.006 0.036	−0.0067 (0.035)	−0.032 (0.095)	−0.026 (0.106)	−1.18E‐11 (0.049)	4.83E‐11 (0.039)
Visuospatial slopes (mean [SD])	−0.034 (0.055)	−0.0387 (0.057)	−0.018 (0.073)	−0.0116 (0.077)	3.94E‐11 (0.032)	2.42E‐11 (0.035)
Global slopes (mean [SD])	−0.027 (0.044)	−0.028 (0.043)	−0.015 (0.065)	−0.0078 (0.055)	1.97E‐11 (0.056)	−1.61E‐11 (0.053)
CDR baseline						
0 (*n*%)	585 (69.64)	677(64.0)	357 (79.2)	213 (72.4)	254 (100)	124(100)
0.5 (*n*%)	255 (30.36)	381(36.0)	94 (20.8)	81(27.6)	0 (0.0)	0 (0.0)
Last CDR						
0 (*n*%)	436 (51.90)	503 (47.54)	296 (65.63)	205 (69.72)	254 (67.19)	124 (32.80)
0.5 (*n*%)	399 (4.75)	546 (51.60)	142 (31.48)	78 (26.53)	‐	‐
≥1 (*n*%)	5 (0.59)	9 (0.85)	13 (2.88)	11 (3.74)	‐	‐
*APOE* ε4 carrier (*n*%)	191 (22.73)	212 (20.03%)	94 (20.84%)	53 (18.02%)	39 (15.35%)	24 (19.35%)
Total females	840 (44.3%)		451 (60.5%)		254 (67.2%)	
Total males		1058 (55.7%)		294 (39.5%)		124 (32.8%)

Abbreviations: *APOE*, apolipoprotein E; CDR, Clinical Dementia Rating; GEM, Gingko Evaluation of Memory; MoVIES; Monongahela Valley Independent Elders Survey; MYHAT, Monongahela‐Youghiogheny Heathy Aging Team; SD, standard deviation.

MYHAT is an ongoing population‐based study that recruited 1982 individuals aged ≥ 65 from southwestern Pennsylvania by age‐stratified random sampling from electoral rolls.^21^ The study excluded individuals who suffered from severe vision or hearing loss, lived in nursing homes at study entry, and/or were decisional impaired. For the present study, we selected the participants who were free of dementia at baseline and were followed annually for the cognitive assessment as described previously.^22^ The MCI/dementia status was determined using CDR values. For the genetic association analyses, we included individuals who had at least two cognitive assessments available during the first 6 years of assessment, from wave 1 to 7. The mean length of follow up was 4.72 years (SD = 1.79, range = 1–6 years, range of assessments/visits = 2–7). From these, we excluded individuals with fewer than two cognitive assessments, those who did not consent to genotyping, individuals who were related within or across the cohorts, and individuals of ancestry other than NHW. This resulted in 745 individuals (451 female and 294 male) available for analysis.

MoVIES was a prospective study that recruited 1681 individuals aged ≥ 65 residing within the Monongahela Valley, Pennsylvania during 1987 through 1989 using a voter's registration list. The individuals did not have severe vision or hearing impairment, were not residing in nursing homes, had at least sixth grade education, and were fluent in English. MoVIES participants were followed every alternate year up to a maximum of 12 years with a mean follow‐up of 11.43 years (SD = 2.15, range = 2–12 years, range of assessments/visits = 2–7).^23^ From the total of 1681 participants, 887 NHWs who were free of dementia throughout the study period were genotyped for the apolipoprotein E (*APOE*) gene.^24^, ^25^ Genome‐wide genotyping was conducted on the 379 participants of the 887 who had sufficient DNA available for analysis. The current analysis includes 378 subjects (254 female,124 male); one was excluded due to the availability of only baseline cognitive data.^22^ CDR values were used to determine diagnosis status in MoVIES. All the MoVIES participants included in this study were free of dementia with CDR of 0 throughout the study.

**RESEARCH IN CONTEXT**

1. **Systematic review**: We reviewed the literature on sex‐specific genetic architecture of cognitive and other complex traits using PubMed. Recent studies have elucidated that sex‐aware analysis can help to identify sex‐specific genetic associations.
2. **Interpretation**: A male‐specific novel association was found nearby *RPS23P3* on chromosome 4 for the decline of memory. A gene involved in oxidative phosphorylation, *NDUFA12*, was found to be associated with the decline of executive function in females but not in males. Several genes (*F7, OAS3, CDH8, ELF5, PIP4K2A*, and *PKIG*) were found to suggestively associate with cognitive decline in a sex‐specific manner. Sex‐stratified analysis is necessary to identify sex‐specific associations and understand the relevant biological pathways.
3. **Future directions**: Future studies based on diverse larger cohorts can help us to understand if these findings are transferable across populations. As our study was based on autosomes, inclusion of the X chromosome can aid in identification of additional genes.

Participants from each study underwent a battery of neurocognitive tests assessing five cognitive domains: attention, executive function, memory, visuospatial function, and language.

### 2.2 Cognitive testing

Detailed descriptions of the cognitive tests across the cognitive domains and the cohorts have been described previously.^26^ The test scores were standardized using the SD and mean. The score was adjusted so that lower scores represents lower cognitive ability. In tests that did not conform accordingly, like Trail Making Tests A and B, the test scores were subtracted from the mean and then divided by the SD. The tests representing specific cognitive domains—based on the theoretical classification of cognitive abilities—were averaged to generate a domain‐specific composite score for individuals who had at least one test score within the domain. A global cognitive score was also generated by averaging all the test scores for individuals who did not miss more than one test.

### 2.3 Modeling phenotype

Individual changes in cognitive domains were calculated by fitting a random slope and intercept linear mixed effect model using age, education,[Table alz14461-tbl-0001] and sex as covariates. Baseline age and education were centered using the mean before being used as covariates. Because the coefficients were slightly left‐skewed due to rapid cognitive decline in some individuals, rank‐based inverse normalization was implemented as described previously.^22^ The slopes were first ranked and scaled (0.1–0.99) and transformed to a standard normal distribution using the qnorm function in R v4.0.0. The cognitive slopes for individuals from the MoVIES cohort were extracted from a previous publication.^22^ The distribution of raw and transformed slopes is presented in Figures S1‐S2 in supporting information.

### 2.4 Genotyping, quality control, and imputation

Individuals were genotyped using DNA from blood from each subject in the cohorts using Illumina Infinium Multi‐Ethnic Global, Illumina Omni2.5, and Omni1‐Quad arrays in GEM, MYHAT, and MoVIES, respectively. Genetic data were filtered to exclude variants with missing call rate > 5% and Hardy‐Weinberg equilibrium *p* < 1E‐06. The subjects self‐reported the ancestry and genetic principal component analysis was also used to assess the self‐reported ancestry. Individuals with genotype missingness > 5%, inconsistent sex, or race were excluded. We checked for overlapping samples and cryptic relatedness within and across cohorts using identity by descent in PLINK^27^ and 23 overlapping and 25 full/half siblings were excluded from the analysis. Imputation was performed using Haplotype Reference Consortium^28^ version 1.1. on the Michigan Imputation Server. Individuals from European White ancestry and variants with minor allele frequency (MAF) ≥ 0.01 and imputation quality *r* ^2^ > 0.3 were retained for analysis. *APOE* genotype was determined by using TaqMan assay or as described previously.^22^, ^29^

### 2.5 Genome‐wide association analyses and genome‐wide meta‐analysis

Genome‐wide association analyses were performed separately for males and females in each cohort examining genetic factors that contribute to the normalized slopes of each cognitive domain and global cognitive function. PLINK was used to conduct linear regression by assuming an additive genetic model. Principal components (PCs) of ancestry were calculated for each cohort and the first four genetic PCs were used as covariates along with age. We conducted a fixed‐effect meta‐analysis on three GWAS of normalized slopes for each domain using standard error–weighted analysis in METAL.^30^ An additional GWAS was conducted to test gene × sex interaction by including the main effects of single nucleotide polymorphism (SNP), sex, and the first four PCs in the linear regression model. Next, we meta‐analyzed the interactive effect of SNP × sex from the three GWAS in each cognitive domain using inverse variance weighted meta‐analysis. The coefficients and the standard error of interaction term were extracted from each study cohort across cognitive phenotypes and fixed‐effect meta‐analysis was conducted using METAL with standard error of coefficients as weights. The suggestive threshold was defined as *p* ≤ 1E‐05 and the genome‐wide significant (GWS) threshold was set to *p* ≤ 5E‐08.

### 2.6 Variant annotation and genetic risk loci characterization

Genetic variants were annotated, and risk loci were characterized using the Functional Mapping and Annotation of Genetic Associations (FUMA).^31^ All variants in linkage disequilibrium (LD; *r* ^2 ^> 0.6) with the top variant within a region were annotated and SNPs within 250 kB of LD blocks were merged into the same locus to define genomic risk loci. FUMA annotates variants for their locations in genes (intronic, exonic, or intergenic) using ANNOVAR, for deleteriousness using Combined Annotation Dependent Depletion (CADD) score, for potential regulatory functions using Regulome DB (RDB) score, and for open chromatin state using 15 categorical states.^32^

### 2.7 Gene mapping

Variants that surpassed the suggestive threshold of significance (*p* ≤ 1E‐05) and were in LD (*r* ^2 ^> 0.6) with the top variant within a region were mapped to genes using positional, expression quantitative trait loci (eQTL), and chromatin mapping in FUMA. SNPs residing within the 10 kB window were mapped. eQTL mapping was conducted using GTEX v8^33^, ^34^ and other brain and blood eQTL databases including BRAINEC,^35^ xQTL,^36^ Van der Wijst,^37^ eQTL Catalogue,^38^ eQTLGen,^39^ and Common Mind Consortium.^40^ Chromatin mapping was conducted to identify if any of the query SNPs interacted with other genetic regions using Hi‐C data from the adult brain cortex.^41^ Along with the mapping via FUMA, we also queried the QTLbase^42^ database. To find the functional relevance of the mapped genes, the GENE2FUNC function in FUMA was used to conduct gene‐enrichment analysis for the genes prioritized via positional, eQTL, and chromatin mapping. Gene Ontology (GO) terms and Kyoto Encyclopedia of Genes and Genomes (KEGG) pathways from the Molecular Signature Database^43^ and the GWAS Catalog were used for the pathway analysis. Benjamini–Hochberg was applied for controlling multiple testing correction and gene sets were considered significant if they surpassed the adjusted false discovery rate of 0.05.

### 2.8 Gene‐based analysis

MAGMA gene and gene‐set analyses were conducted in FUMA. For the gene‐based analyses, the gene‐wide threshold was determined as *p* = 2.762E‐06 (0.05/18107 genes). For the MAGMA gene‐set analysis, the curated gene sets and GO terms from Molecular Signature Databases were used.^43^ Bonferroni correction was applied to gene‐set analyses.

## 3 RESULTS

Table 1 presents the sex‐stratified data on age, education, and five cognitive domains along with global cognitive function derived from five domains in the three cohorts. The total number of female and male participants from three cohorts was 1545 and 1476, respectively. Female participants were slightly older than male participants in MoVIES. Male participants were slightly better educated than female participants in all three cohorts.

### 3.1 Genome‐wide meta‐analysis

**FIGURE 1:**
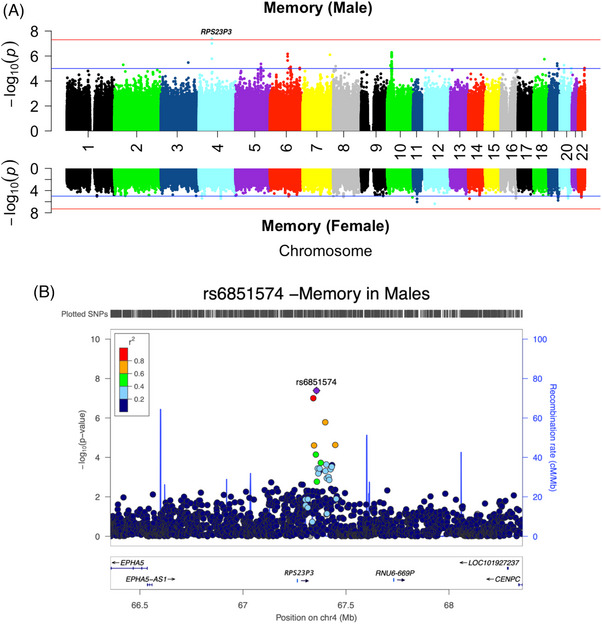
A, Miami plots of sex‐stratified meta‐analyses for memory slopes in male(top) and female (bottom) participants. The blue line indicates a suggestive threshold of *p* ≤ 1E‐05 and the red line indicates the threshold at *p* = 5E‐08. B, Locus zoom plot around the top SNP rs6851574 in male participants. GWS, genome‐wide significant; SNP, single nucleotide polymorphism

Sex‐stratified quantile–quantile (QQ) plots and the genomic control factor (*λ*) did not show signs of inflation in any of the cognitive domain analyses (Figure S3 in supporting information). The previously reported novel GWS association of rs6559700 on chromosome 9 in this sex‐combined sample with the decline of attention (*p* = 2.69E‐08, *β* = −0.23)^26^ was found to be stronger in women (*p* = 6.21E‐07, *β* = −0.28) than men (*p* = 4.94E‐03, *β* = −0.175), but there was no SNP × sex interaction (*P*_interaction_ = 0.19). Similar results were obtained for the established association of *APOE* ε4 with the decline of memory in the sex‐combined (*p* = 5.58E‐09, *β* = −0.225), female (*p* = 1.6E‐04, *β* = −0.207) and male (*p* = 3.9E‐06, *β* = −0.255) samples. In the current sex‐stratified genome‐wide meta‐analysis, we identified a novel GWS association of rs6851574 on chromosome 4 with the decline of memory in male participants (*P*_male_ = 4.10E‐08, *β*_male_ = 0.19; *P*_female_ = 6.13E‐01, *β*_female_ = 0.017; *P*_interaction_ = 3.76E‐04) but not in female participants (Figure 1). The top SNP was 59,691 bp downstream of *RPS23P3* (ribosomal protein S23 pseudogene 3). The direction of the effect of the minor allele was consistent in all three cohorts (Table 2). *RPS23P3*/rs6851574 was also nominally significant in male participants but not in female participants for decline of global function (*P*_male_ = 1.87E‐05, *β*_male_ = 0.149; *P*_female_ = 7.93E‐01, *β*_female_ = −0.009), visuospatial function (*P*_male_ = 1.11E‐02, *β*_male_ = 0.094; *P*_female_ = 6.00E‐01, *β*_female_ = −0.018), and language (*P*_male_ = 2.50E‐02, *β*_male_ = 0.080; *P*_female_ = 9.52E‐01, *β*_female_ = −0.002; see also Table S1). We further conducted a linear regression on the top SNP using an additive model to understand if *APOE* ε4 was confounding the interaction among sexes. The decline of memory was used as an outcome and the effect of SNP × sex was tested by adjusting for sex, study cohort, and the presence of *APOE* ε4. rs6851574 was nominally significant even after adjusting for *APOE*4 (*p* = 6.96E‐04). *RPS23P3*/rs6851574 remained GWS when it was regressed with the decline of memory in males after adjusting for the study cohort and the presence of *APOE*4 (*p* = 4.66E‐08).

**TABLE 2:** List of top and suggestive SNPs with *p* < 1E‐06 associated with each neurocognitive domain and global cognitive function.

Domain	Sex	SNP	Chr	A1/A2	Loc	Gene Region	Meta‐analysis				*P*_other sex_	*P*_Interaction_	GEM			MYHAT			MoVIES		
							MAF	*β*	*p*	Dir			MAF	*β*	*p*	MAF	*β*	*p*	MAF	*β*	*p*
**Memory**	**M**	**rs6851574**	**4**	**G/T**	**intergenic**	***MIR1269A, LOC101927237***	**0.39**	**0.19**	**4.100E‐08**	**+ + +**	6.13E‐01	3.76E‐04	0.39	0.18	1.87E‐05	0.39	0.24	2.00E‐03	0.38	0.20	1.22E‐01
		rs76585043	10	A/G	intronic	*PIP4K2A*	0.17	−0.23	5.130E‐07	‐ ‐ ‐	6.22E‐02	4.11E‐07	0.16	−0.21	7.04E‐05	0.18	−0.28	5.25E‐03	0.20	−0.23	1.85E‐01
		rs4706392	6	T/A	intergenic	*MAP3K7, MIR4643*	0.19	0.21	6.780E‐07	+ + +	4.68E‐01	4.30E‐03	0.19	0.23	1.17E‐05	0.20	0.16	7.76E‐02	0.20	0.24	1.12E‐01
		rs6943682	7	A/G	intergenic	*TRY2P, MIR11400*	0.17	−0.22	7.950E‐07	‐ ‐ ‐	2.42E‐01	2.55E‐05	0.16	−0.25	3.97E‐06	0.20	−0.11	2.53E‐01	0.18	−0.28	6.39E‐02
	**F**	rs10587831	12	C/T	intronic	*KRT8*	0.23	−0.27	4.22E‐07	‐ ‐ ‐	4.11E‐01	2.77E‐02	0.03	−0.11	3.63E‐01	0.29	−0.29	4.12E‐06	0.03	−0.61	9.06E‐03
**Attention**	**F**	rs73490304	18	A/T	intergenic	*GALR1, LINC01029*	0.08	0.33	2.69E‐07	+ + +	4.31E‐01	4.53E‐05	0.07	0.30	6.44E‐04	0.09	0.22	6.26E‐02	0.10	0.55	1.57E‐04
		rs6532920	4	A/G	intronic	*PPP3CA*	0.32	−0.18	4.20E‐07	‐ ‐ ‐	5.92E‐01	8.17E‐05	0.32	−0.22	4.00E‐06	0.33	−0.17	1.75E‐02	0.32	−0.07	4.17E‐01
		rs6559700	9	A/G	intergenic	*RASEF*, *FRMD3*	0.10	−0.28	6.21E‐07	‐ ‐ ‐	4.95E‐03	1.91E‐01	0.11	−0.26	3.55E‐04	0.10	−0.26	1.50E‐02	0.10	−0.38	9.62E‐03
**Executive**	**F**	**rs11107823**	12	A/G	intergenic	*KRT19P2, NDUFA12*	0.12	0.28	**9.35E‐08**	+ + +	1.51E‐01	7.42E‐06	0.12	0.26	0.000259	0.12	0.21	2.71E‐02	0.10	0.54	2.02E‐04
		rs78332132	2	A/G	intergenic	*GYPC, TEX51*	0.06	0.36	3.98E‐07	+ + +	3.08E‐02	1.82E‐07	0.06	0.28	1.99E‐03	0.06	0.53	8.14E‐05	0.05	0.34	1.04E‐01
	**M**	rs7580515	2	G/A	intergenic	*GPD2, GALNT5*	0.49	0.17	8.120E‐07	+ + +	1.43E‐01	4.19E‐06	0.49	0.18	1.57E‐05	0.48	0.06	4.21E‐01	0.52	0.32	3.05E‐03
**Language**	**M**	rs77245822	5	G/A	intronic	*PDZD2*	0.08	0.32	4.240E‐07	+ + +	7.84E‐01	2.07E‐04	0.08	0.31	4.10E‐05	0.07	0.14	3.86E‐01	0.10	0.56	9.19E‐04
		rs7204089	16	A/G	intronic	*CDH8*	0.07	−0.33	9.120E‐07	‐ ‐ ‐	5.95E‐02	2.74E‐02	0.07	−0.33	4.29E‐05	0.07	−0.29	5.27E‐02	0.08	−0.40	5.34E‐02
**Visuospatial function**	**M**	rs10486576	7	C/T	intronic	*JAZF1*	0.12	0.28	3.81E‐07	+ + +	5.65E‐01	5.85E‐06	0.12	0.20	1.65E‐03	0.10	0.53	1.20E‐04	0.13	0.47	9.55E‐03
		rs11791658	9	G/A	intronic	*EHMT1*	0.28	0.20	4.43E‐07	+ + +	5.60E‐01	5.75E‐04	0.27	0.18	6.73E‐05	0.29	0.20	1.86E‐02	0.28	0.33	2.81E‐02
		rs12594684	15	T/C	intronic	*BUB1B‐PAK6, PAK6*	0.01	0.73	7.92E‐07	+ + +	5.17E‐01	6.94E‐04	0.01	0.88	3.69E‐07	0.02	0.25	4.78E‐01	0.02	0.45	3.81E‐01
**Global** function	**F**	rs146166019	6	T/G	intergenic	*MIR3668, MIR4465*	0.02	0.64	5.57E‐07	+ + +	6.47E‐03	6.58E‐08	0.02	0.70	2.63E‐05	0.02	0.55	2.02E‐02	0.01	0.55	1.43E‐01
	**M**	rs6496242	15	A/G	intergenic	*LOC101927310, LINC00923*	0.17	−0.22	7.90E‐07	‐ ‐ ‐	4.36E‐01	1.09E‐04	0.17	−0.22	4.91E‐05	0.18	−0.22	2.57E‐02	0.19	−0.23	9.65E‐02
		rs12204948	6	C/A	intronic	*ARMC2*	0.16	0.23	9.44E‐07	+ + +	1.04E‐01	2.86E‐02	0.16	0.21	1.72E‐04	0.17	0.33	9.14E‐04	0.12	0.10	5.76E‐01

Abbreviations: A1, minor allele; A2, major allele; Chr, chromosome; GEM, Gingko Evaluation of Memory; MAF, minor allele frequency; MoVIES, Monongahela Valley Independent Elders Survey; MYHAT, Monongahela‐Youghiogheny Heathy Aging Team; SNP, single nucleotide polymorphism.

**FIGURE 2:**
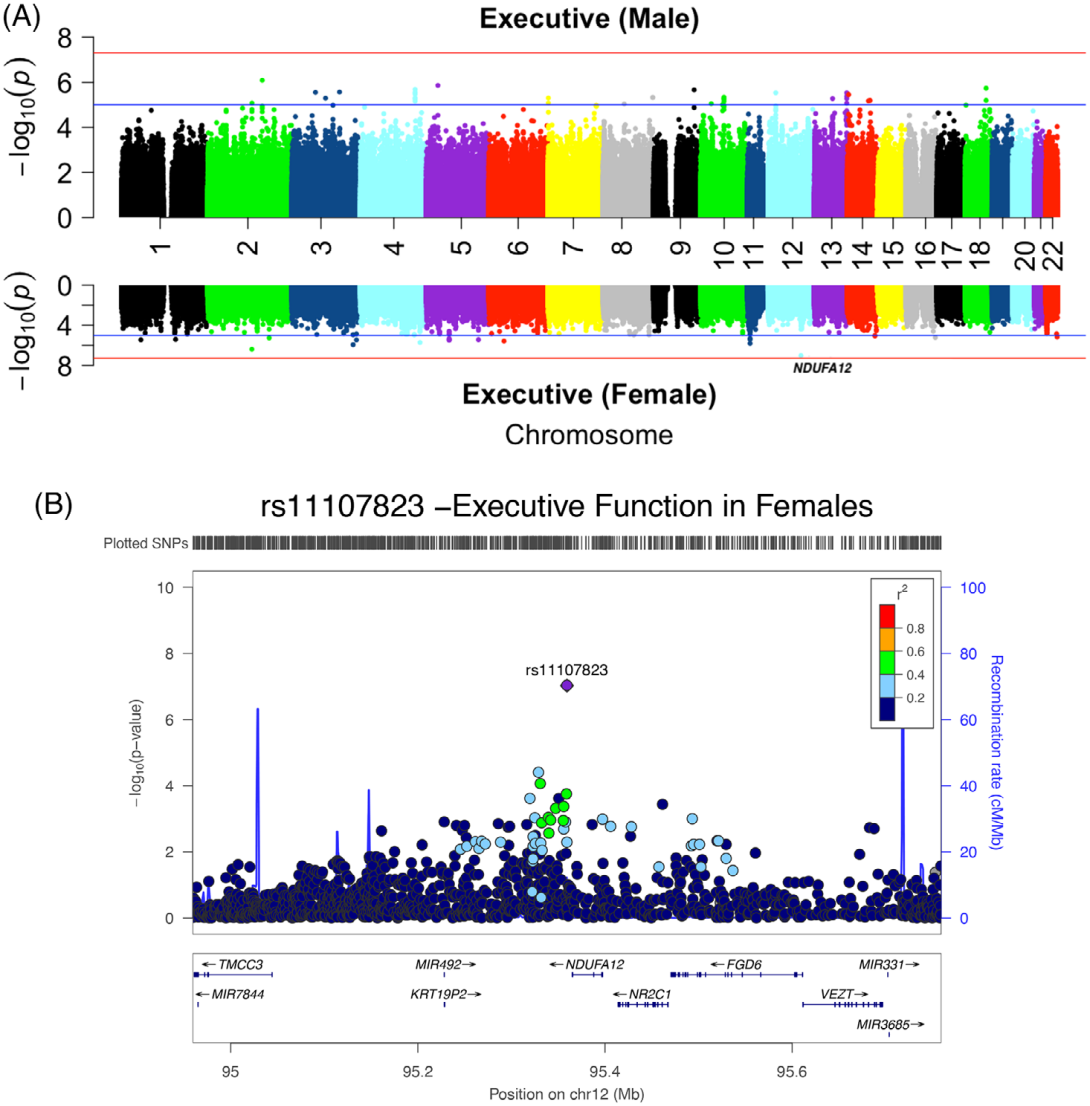
A, Miami plots showing the genome‐wide meta‐analysis of sex‐stratified analysis of slopes of executive functioning in male participants (top) and female participants (bottom). The blue line indicates a suggestive threshold of (1E‐05), and the red line indicates the GWS threshold line at *p* = 5E‐08. B, Locus zoom plot of the SNP rs11107823 in female participants. GWS, genome‐wide significant; SNP, single nucleotide polymorphism

We also identified a subthreshold GWS locus, rs11107823, for the decline of executive function on chromosome 12, in female participants but not in male participants (*P*_female_ = 9.35E‐08, *β*_female_ = 0.28; *P*_male_ = 0.1509, *β*_male_ = −0.077; *P*_interaction_ = 7.421E‐06). The top SNP is in the intergenic region near *NDUFA12* (Figure 2). The direction of the effect of the minor allele was consistent in all three cohorts (Table 2). *NDUFA12*/rs11107823 was also nominally associated with global cognitive decline in females (*P*_female_ = 8.63E‐04, *β*_female_ = 0.187; *P*_male_ = 0.940) as shown in Table S1 in supporting information. We tested for the SNP × sex interaction by regressing the executive function slopes against the copies of minor alleles of SNPs adjusting for sex, study cohort, and presence of *APOE*4 by using an additive model. rs11107823 was found to have a significant differential effect (*p* = 2.12E‐06) on the sexes even after adjusting for the effect of *APOE*4. We also tested if the protective effect of *NDUFA12*/rs11107823 on the decline of executive function was significant in females participantsadjusting for the presence of an *APOE*4 allele and study cohort, and found the SNP to have a significant protective effect in females (*p* = 1.07E‐07).

To test if the observed associations were driven by incident MCI/dementia, we also conducted meta‐analyses on the top male‐ and female‐specific SNPs using the final visit CDR (0, 0.5, and ≥ 1) as an additional covariate. After the adjustment, both top SNPs showed similar allelic effects and significance: rs6851574 with the decline of memory in male participants (*p* = 1.81E‐07, *β* = 0.17); rs11107823 with the executive function in female participants (*p* = 3.63E‐08, *β* = 0.28), confirming that these associations are driven by cognitive decline.

In addition to the two associations, we observed 17 suggestive associations at *p* < 1E‐06 (range 2.69E‐07–9.44E‐07) with consistent directions of the effect of the minor alleles in all three cohorts (Table 2). Notably, *JAZF1*/rs10486576, which was associated with the decline of visuospatial function in male participants (*p* = 3.81E‐07; *P*_sex‐interaction_ = 5.85E‐06), is a recently identified AD risk gene.^44^ The sex‐stratified list of suggestive SNPs (*p* < 1.0E‐05) and their associations with cognitive domains is shown in Tables S2–S13 and Figures S4–S6 in supporting information. rs35322174, an intergenic SNP at *BIN1/CYP27C1*, was also found to be suggestively associated with decline of executive function in male participants (*p* = 8.41E‐06, *β* = −0.16; Table S9). The SNP is not in LD with the known AD risk variant rs6733839 at the *BIN1* locus.

### 3.2 Gene‐based analysis

We performed MAGMA gene‐based via FUMA to identify genes that are implicated in cognitive decline across the cognitive domains. Although none of the genes surpassed the gene‐wide threshold of *p* = 2.762E‐06 (0.05/18107), several genes were implicated through single‐variant as well as gene‐based analysis. The top five genes in each domain are included in Table S14 in supporting information. We focused on the genes that were suggestively associated in both single‐variant and gene‐based analysis and were functionally relevant to cognitive decline. In males, this included *F7* associated with decline of executive function (*P*_gene_ = 1.36E‐05)*, ELF5* associated with the decline of global cognitive function (*p* = 2.05E‐05)*, OAS3* associated with decline of attention (*P*_gene_ = 2.58E‐05) and *PIP4K2A* (*P*_gene_ = 7.28E‐05), and *APOC1* (*p* = 1.52E‐04) associated with decline of memory, *CDH8* associated with the decline of language (*P*_gene_ = 1.70E‐04). In females participants, these genes included *PKIG* associated with the decline of memory (*P*_gene_ = 3.24E‐05) and global function (*P*_gene_ = 6.69E‐05) and *TRIML2* associated with the decline in global cognitive function (*P*_gene_ = 2.36E‐05). The gene *NDUFA12*, which was in eQTL for the sentinel SNP rs1107823, and was associated with the decline of executive function in female participants, was also nominally associated with the decline of executive function in female participants (*p* = 1.40E‐04) in the gene‐based analysis. We performed the MAGMA gene‐set analysis using the GO terms and curated gene sets from Molecular Signature Database v6.1 in FUMA. GATOR1 complex was enriched for the decline of language in females (*p* = 0.049) and delacroix_rar_targets_dn (regulation of target genes by retinoic acid) was enriched for the decline of the visuospatial function in male participants (*p* = 0.004). The enriched pathways are represented in Table S14. All other cognitive phenotypes showed no enrichment in MAGMA gene‐set analysis.

### 3.3 Gene mapping

The variants that surpassed the suggestive genome‐wide threshold of *p* = 1E‐05 and those in LD were used for conducting gene mapping in FUMA to identify genes involved in cognitive decline (Tables S15–S26 in supporting information). Positional mapping matches the SNPs to genes based on physical location, eQTL mapping annotates the SNP to a gene if the variant is found to alter the gene expression, and chromatin mapping matches SNPs to genes if any of the query SNPs are found to interact with the other genes in spatial conformation. We used the Hi‐C data from the adult human cortex to conduct the chromatin mapping. Along with FUMA, we also used QTLbase to find quantitative trait loci (QTLs) for the top SNPs. The lead SNP, rs11107823, associated with decline of executive function in female participants, was found to be an eQTL for *NDUFA12* (*p* = 0.0031, *β* = 0.4838), *NTN4* (*p* = 0.0003, *β* = 0.616) in blood macrophages, for *NR2C1* in blood monocytes (*p* = 0.011), and for *VETZ* in brain prefrontal cortex (*p* = 0.0043, *β* = −0.0264). The following genes were mapped using positional, eQTL, and chromatin mapping in female participants: *COPA* (attention), *PPFIBP2* (executive function), *ALDH16A1*, *PKIG* (memory), *DDX46*, *PCBD2*, *C20orf24*, *NDRG3* (language), and *ADTRP* (global function). Likewise, the following genes were mapped in male participants: *PTPRN2*, *IL32*, *ZNF213* (attention), *PDSS2, CTB‐129P6.4* (memory), *KLF7*, *KLF7‐IT* (language), and *ARMC2* (global function). The list of the mapped genes in each domain across sexes is provided in Tables S27–S38 in supporting information.

To understand if the cognitive domains have shared genetic influence, we queried if the same genes were mapped across two or more cognitive phenotypes. We found that *RRAGA*, *PLIN2*, *RP11‐146N23.1*, and *DENND4C* were mapped for the decline of attention and global function in female participants. Keratin genes clustered on chromosome 12q12‐q13 (*KRT5*, *KRT72*, *KRT73*, *KRT1*, *KRT3*, *KRT79*, *KRT78*, *KRT8*, and *KRT18);* along with *NAV2*, *RPL7P41*, *NR4A1*, *AC107016.2*, *AC107016.1*, and *EIF4B* mapped for the decline of memory and global function in females. In males, *snoU13* was mapped for the decline of attention and language. Similarly, *PTPRN2*, *NCAPG2*, *ESYT2*, and *WDR60* were mapped for the decline of attention and visuospatial function. *PDSS2* was mapped for the decline of memory and language in males. A ribosomal pseudo gene, *RPL17P12*, and an RNA pseudo gene, *RNU7‐2P*, were mapped for the decline of visuospatial function and global function in male participants. *ANGPT1* was found to be associated with the decline of language in both males and female participants.

### 3.4 Gene‐enrichment analysis

The list of mapped genes was further used to perform gene‐set enrichment analysis using the GENE2FUNC function in FUMA. GO terms and KEGG pathways from the Molecular Signature Database and the GWAS Catalog were used for the pathway analysis. Men and women had differing enrichment pathways and traits associated across the cognitive domains and global cognitive function (Table S39 in supporting information). Genes enriched for the decline of attention in females were associated with cellular transport of ions, cations, and lipid localization, while in males these were related to drug and ribonucleotide binding activities. The decline of memory and global function in women was enriched for keratin gene‐related pathways such as cornification and keratinization, while in male participants, memory function was enriched for the genes related to immune response and AD phenotypes. Genes mapped for the decline of executive function and visuospatial functions in men were enriched for genes associated with blood clotting factors, erectile dysfunction, and anorexia nervosa, respectively; while in females, these were associated with calcium ion binding, empathy quotient, and severity of herpes virus, respectively.

### 3.5 Cognitive‐associated SNPs with AD risk

To understand whether these cognitive‐associated SNPs are also associated with AD risk, we queried the *p* values of the cognition‐associated SNPs in the discovery dataset of the International Genomics of Alzheimer's Project (IGAP) comprising ≈ 64,000 subjects.^45^ Fourteen SNPs that were suggestively associated with attention, memory, and global function in women were found to be nominally associated with AD risk, with *p* values ranging from 0.0039 to 0.036 (Table S40 in supporting information). Similarly, 19 variants associated with cognitive function in men were found to be nominally associated with AD, with *p* values ranging from 1.63E‐03 to 4.85E‐02 (Table S41 in supporting information).

### 3.6 AD‐associated SNPs with cognitive decline

We also queried the association of top reported AD variants^44^ in our cognitive phenotypes in both males and females. Of the 98 reported variants, 53 were present in our datasets. *APOE*4/rs429358 showed the most significant association among all inquired variants; it was associated in both sexes with memory (*P*_male_ = 4.0E‐06, *P*_female_ = 1.6E‐04), language (*P*_male_ = 5.8E‐03, *P*_female_ = 1.4E‐03), and global function (*P*_male_ = 1.86E‐05, *P*_female_ = 9.81E‐05) and in males only with executive function (*P*_male_ = 1.4E‐04, *P*_female_ = 0.07). *IL34/*rs4985556 was associated nominally in females with memory (*P*_female_ = 8.9E‐03), language (*P*_female_ = 0.034), and global function (*P*_female_ = 0.044). Altogether, 9 and 15 AD‐associated variants were found to be nominally associated with cognitive slopes in females (Table S42 in supporting information) and males (Table S43 in supporting information), respectively.

## 4 DISCUSSION

In the present study, we investigated the sex‐specific genetic architecture of cognitive decline across multiple cognitive domains and global cognitive function in 1545 female and 1476 male participants derived from three longitudinal cohorts. Intra‐individual cognitive slopes over time were derived within each cohort for the attention, executive function, memory, language, and visuospatial domains, and for global cognitive function. We conducted genome‐wide meta‐analyses and gene‐based and gene‐set analyses to identify potential differential genetic effects between the sexes.

**FIGURE 3:**
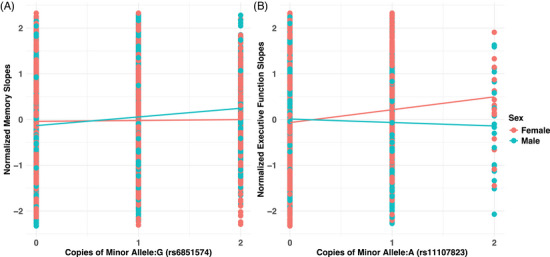
Illustration of gene x sex interaction of RPS23P3/rs6851574 on the decline of memory (A) and NDUFA12/rs11107823 on the decline of executive function (B). The increase in the number of the G allele of RPS23P3/rs6851574 is associated with protection against the decline of memory in male participants but not in female participants. Similarly, the increase in the number of A alleles of NDUFA12/rs11107823 is associated with protection against the decline of executive function in women.

Our genome‐wide meta‐analyses identified two novel associations, one in men and one in women (Figure 3). The novel men association of rs6851574 was observed with the decline of memory in the intergenic region near *RPS23P3* on chromosome 4 (*P*_male_ = 4.10E‐08, *β* = −0.19). Although the functional role of rs6851574 is not yet known, this intergenic region has been previously associated with several brain and cognition‐related traits, including anatomical brain aging,^46^ brain shape,^47^ and educational attainment.^48^ *RPS23P3* is a ribosomal protein 23 pseudogene 3 and *RNU6‐699P* represents RNA, U6 small nuclear 699. Pseudogenes, one thought to be non‐functional, may regulate gene expression by producing interfering RNA sequences that can bind to and silence mRNA.^49^

The novel female‐specific subthreshold association of *NDUFA12*/rs11107823 was observed on chromosome 12, where the minor allele was found to confer protection against the decline of executive function (Figure 3). The rs11107823 SNP lies 5763 bp upstream of *NDUFA12* and was found to be an eQTL for several nearby genes, including *NDUFA12*, *VETZ*, *NR2C1*, and *NTN4* in blood and brain tissues. In the gene‐based analysis, *NDUFA12* was found to be one of the top genes associated with the decline of executive function in females (*p* = 1.4E‐04). *NDUFA12* codes for the supernumerary subunit A12 of mitochondrial complex I, the largest oxidative phosphorylation complex. Complex I establishes the gradient for the generation of adenosine triphosphate by transferring protons from nicotinamide adenine dinucleotide to ubiquinone. Loss‐of‐function mutations in *NDUFA12* are known to cause Leigh's syndrome, a neuromuscular disorder characterized by central nervous system lesions and progressive loss of psychomotor functions in infants, due to disruption of complex I.^50^, ^51^ A study on gene expression differences in the temporal and prefrontal brain regions of both sexes across ages found that energy production and metabolism‐related genes were downregulated in aging males but not in females.^19^ Another study showed that women have higher mitochondrial content and functional capacity in both peripheral blood and the brain.^52^ The downregulation of oxidative phosphorylation genes contributes to aging and AD, as altered glucose metabolism is a feature of AD.^53^, ^54^ A study comparing cell type–specific transcriptomic changes in the cortex of AD patients to those of normal controls found that the expression of respiratory genes along with *NDUFA12* was downregulated in inhibitory neurons in AD patients.^55^ Although it is not clear why women tend to be disproportionally affected with dementia despite having enhanced mitochondrial function and energy metabolism, these findings suggest that enhanced mitochondrial function and energy generation confer a female advantage against cognitive decline in aging.

We prioritized genes identified through both single‐variant and gene‐based analyses. *F7* that showed association with executive function in men was previously associated with cognitive impairment.^56^ *OAS* and *ELF5* that showed association with attention and global function, respectively, in mens have been implicated with COVID‐19 severity.^57^, ^58^, ^59^ *ELF5* is differentially regulated in aged microglia^60^ and contributes to accelerated brain aging through immunomodulation.^61^ *CDH8* (language‐associated in males) encodes a crucial protein for the assembly of corticostriatal synapses and circuits and is a known risk gene for autism spectrum disorder.^62^, ^63^, ^64^ *PIP4K2A* (memory‐associated in male participants) encodes a lipid kinase that phosphorylates phosphatidylinositol‐5‐phosphate, a substrate of the phosphoinositide signaling pathway, regulating cellular functions like motility, membrane trafficking, and cytoskeletal organization.^65^ *PIP4K2A* is shown to interact with phosphorylated tau in the brain of AD cases.^66^ *PKIG* (memory and global function–associated in female participants) codes for the protein kinase inhibitor of c‐AMP‐dependent protein kinase, which plays a vital role in learning and memory.^67^

From the gene‐set enrichment analysis, we found that biological pathways relating to cognitive endophenotypes differ across sexes. MAGMA gene‐set enrichment analysis revealed that the GATOR1 complex is associated with decline of language in females, while retinoic acid receptor (RAR) targets are associated with visuospatial function decline in males. While RAR is involved in synaptic plasticity, and affects learning and memory, GATOR1 regulates mTORC1, a pathway tied to aging, cancer, and other diseases.^68^, ^69^, ^70^ Similarly, gene‐set enrichment analysis using variants that surpassed suggestive thresholds also found that membrane transport, including both ion transport and lipid transport, was enriched for the decline of attention in females. Studies have shown that membrane transport and lipid metabolism play a role in regulating aging and longevity^71^, ^72^ and these effects also vary across sexes.^73^ Interestingly, the decline of memory and global cognition in female participants was enriched by biological processes such as intermediate filament, cornification, and keratinization. It is noteworthy that one of the keratin genes, *KRT8*, which was also suggestively associated with decline of memory in females, was found to promote amyloid fibril formation in the liver in alcoholic liver disease.^74^ However, the precise role of these structural proteins in cognitive decline remains elusive. The incorporation of epigenetic effects, the regulatory influence of sex hormones, and other environmental factors could help us understand the differences in the biology of cognitive aging across sexes.

We conducted meta‐analyses of cognitive phenotypes across five cognitive domains built on comprehensive neuropsychological tests and global cognitive function from three longitudinal cohorts of cognitive decline. Although several prior studies have focused on sex‐specific cognitive phenotypes, these were limited to one or a few domains. This is the first study to conduct sex‐specific meta‐analyses across five cognitive domains and global cognition in three independent older cohorts, offering a broader and deeper perspective of cognitive decline in older adults. Conducting single‐variant, gene‐based, and gene‐mapping analyses has allowed us to identify putative candidate genes across domains. The association of *APOE* variants with several cognitive domains in both males and females in this study reassures the validity of our findings. Furthermore, our study has 90% power to detect 2.98% and 2.85% variance in men and women, respectively, at GWS level explained by a single SNP.

However, our study also has limitations. Multiple methods are available for genome‐wide associations of longitudinal cognitive scores. Our study assumed that cognitive decline follows a linear trajectory across time, but we recognize cognitive change across time could also involve non‐linear trends. Although we found robust associations of *APOE* ε4 and other top SNPs while comparing the slope‐based method with linear mixed effect modeling and generalized estimating equations, genome‐wide associations of longitudinal cognitive scores can give different outcomes based on the methods used. Additionally, we used *z* score–based methods to derive the slopes, but this approach has limitations: within domains, different tests likely have different psychometric properties (e.g., difficulty, reliability, ceiling/floor effects, and association with the underlying construct), which *z* scoring does not address. Alternative methods such as modern psychometric techniques could improve the precision of the cognitive phenotypes.^75^ Thus, it is important to assess the utility and reliability of different methods in multiple larger longitudinal cohorts. Although we have investigated the sex‐specific genetic architecture of cognitive decline across sexes, our study is limited to the genetic variation in autosomal chromosomes, and the effect of X chromosomes on the genetic architecture of cognition across sexes remains to be explored. Including X chromosomal variation may help to identify additional genes and pathways to understand sex‐specific cognitive decline in greater detail. Also, as our study was based on NHWs, these findings may or may not generalize to other populations. Further studies are required to replicate these findings on larger cohorts across more diverse populations and functionally validate the effect of these loci on cognitive decline.

Our results suggest that there are sex‐specific associations across the cognitive domains in older adults. We identified a novel GWS association on chromosome 4q13.2 with the decline of memory in males and a subthreshold GWS association on chromosome 12q22 for the decline of executive function in female participants. The gene‐set enrichment analysis also suggests there is variability in enriched biological pathways and traits across sexes in cognitive changes across cognitive domains and global cognitive function. Attention decline in female participants was associated with membrane transport, while memory and global cognitive decline were associated with keratinization and epithelial cell formation genes. In male participants, the mapped genes for the memory decline were associated with adaptive immune response and AD‐related traits, and mapped genes for the decline of executive function were associated with factor VII levels. Our work provides a framework for the replication of these findings in larger and independent cohorts, which was a limitation in this study, and further investigation of differential effects of biological processes across sexes. Sex‐aware genetic studies could provide novel insights into cognitive decline and inform sex‐specific pharmacological approaches in healthy aging as well as other late‐onset diseases in which cognitive abilities are impaired. Future work should also incorporate X chromosomes to provide the complete genetic architecture of cognitive decline in men and women.

## CONFLICT OF INTEREST STATEMENT

Dr. DeKosky is an associate editor of *Neurotherapeutics*, the official journal of the American Society for Experimental Neurotherapeutics. He also serves on the medical advisory board for Acumen Pharmaceuticals, Cognition Therapeutics, and Vaccinex and on the data safety and monitoring board of Biogen and Prevail Pharmaceuticals. Dr. DeKosky is also a consultant at Boxer Capital, Brainstorm Cell Therapeutics, Lundbeck Pharmaceuticals, Amylyx Pharmaceuticals, Reata Pharmaceuticals, and Biogen. Dr. Lopez also serves on the consulting committee for Biogen, Novo Nordisk, EISAI, and Lundbeck. The submitted work is unrelated to any of these. All other authors declare no conflict of interest. Author disclosures are available in the supporting information.

## CONSENT STATEMENT

Written informed consent was obtained from all participants included in the study in accordance with the University of Pittsburgh Institutional Review Board.

## Supporting information



Supporting Information

Supporting Information

Supporting Information
