# Supplementary material for: Sex‐stratified genome‐wide meta‐analysis identifies novel loci for cognitive decline in older adults
Source: Alzheimers Dement. 2025 Mar 5;21(3):e14461. doi: 10.1002/alz.14461 (PMC11880917; doi:10.1002/alz.14461)
Supplement: Supplementary file 2 — Supporting Information [file ALZ-21-e14461-s001.docx]

**Supplementary Material**

**Sex-stratified genome-wide meta-analysis identifies novel loci for cognitive decline in older adults**

Vibha Acharya^a^, Kang-Hsien Fan^a^, Beth E. Snitz^b^, Mary Ganguli^b,c,d^, Steven T. DeKosky^d^, Oscar L. Lopez^b^, Eleanor Feingold^a^, M. Ilyas Kamboh^a,c^

^a^Department of Human Genetics, University of Pittsburgh School of Public Health, Pittsburgh, PA 15261, USA

^b^Department of Neurology, School of Medicine, University of Pittsburgh, Pittsburgh, PA 15213, USA

^c^Department of Psychiatry, School of Medicine, University of Pittsburgh, Pittsburgh, PA 15213, USA

^d^Department of Epidemiology, University of Pittsburgh School of Public Health, Pittsburgh, PA 15261, USA

^e^McKnight Brain Institute and Department of Neurology, College of Medicine, University of Florida, Gainesville, FL 32610, USA


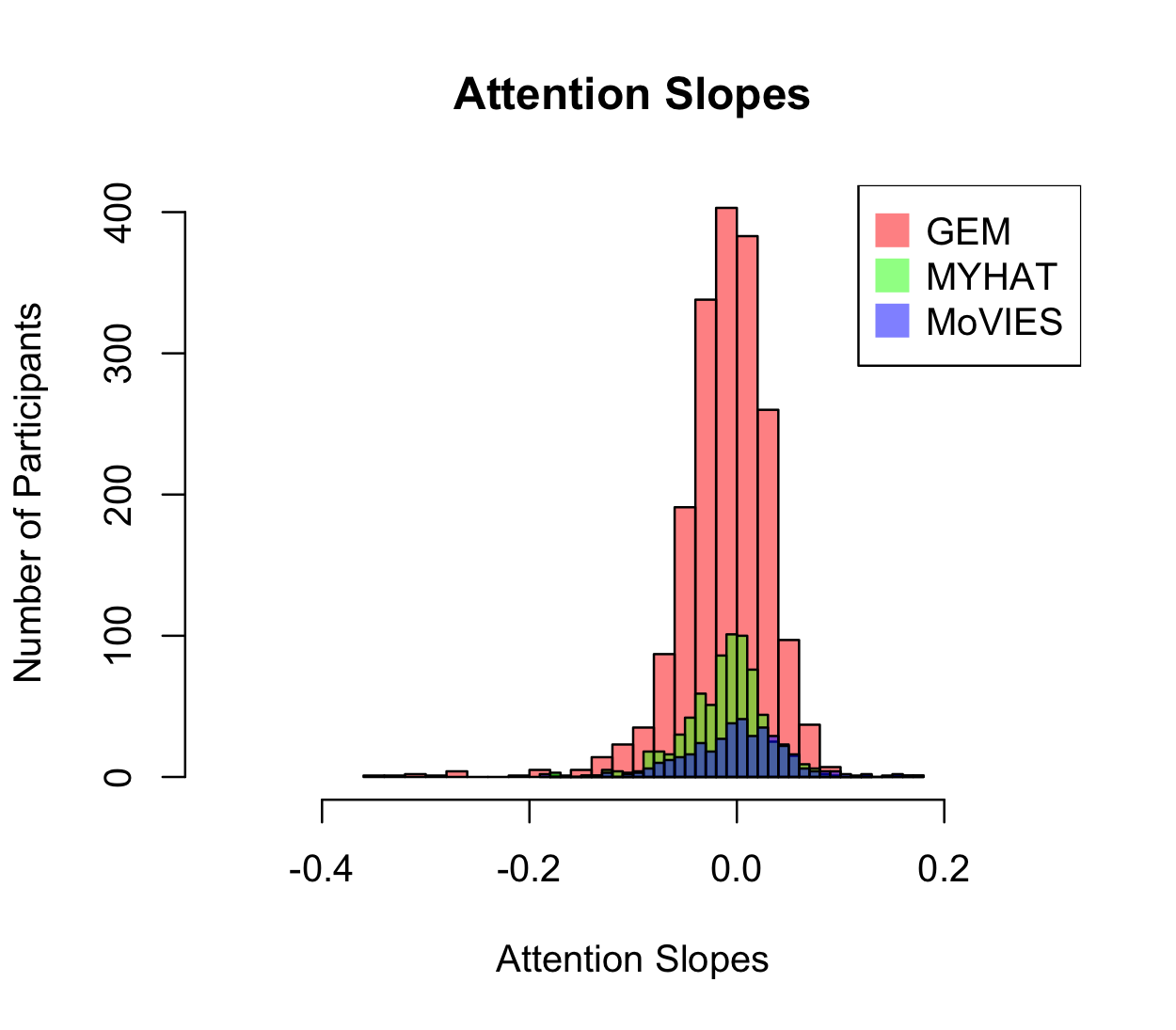

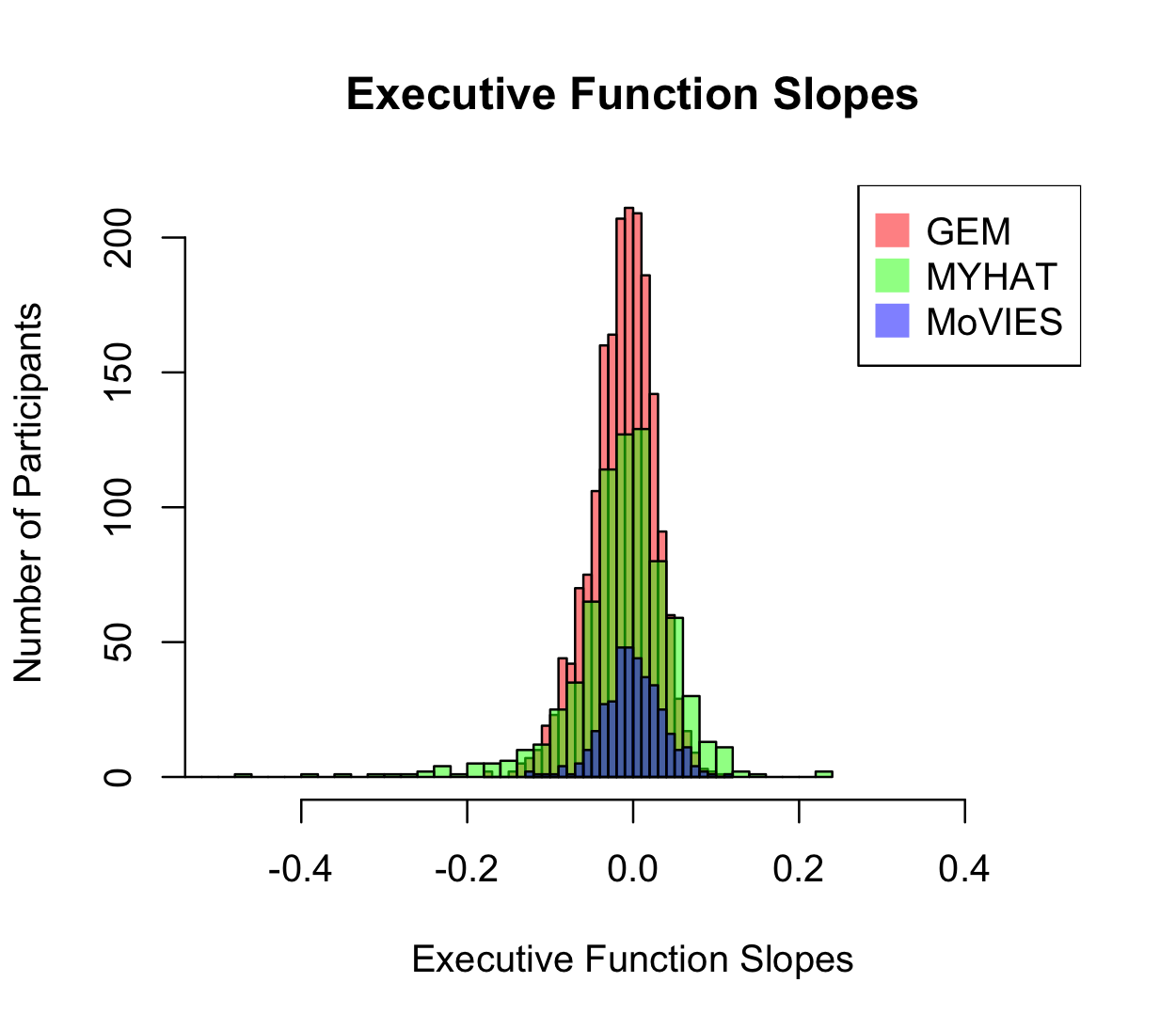

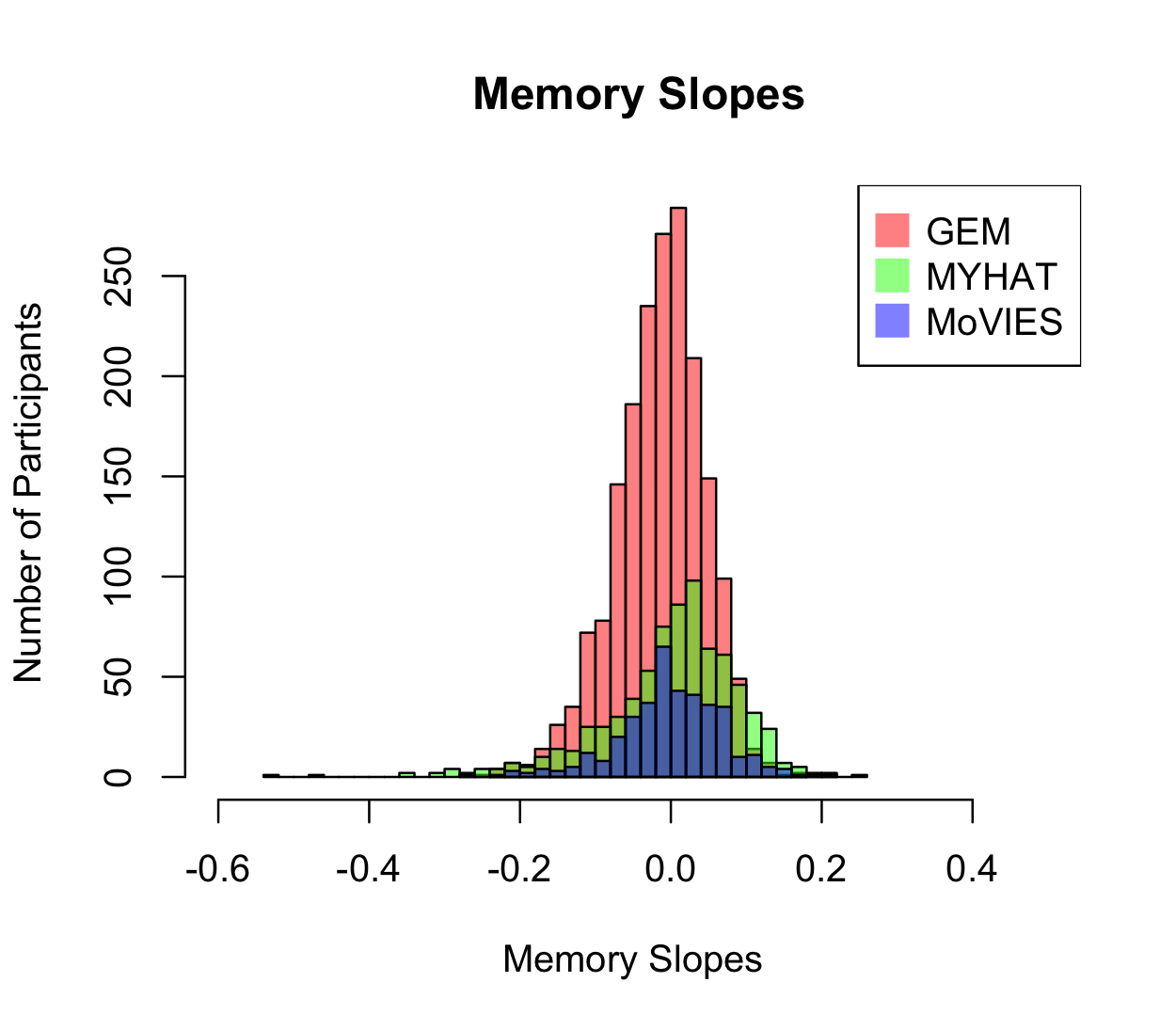

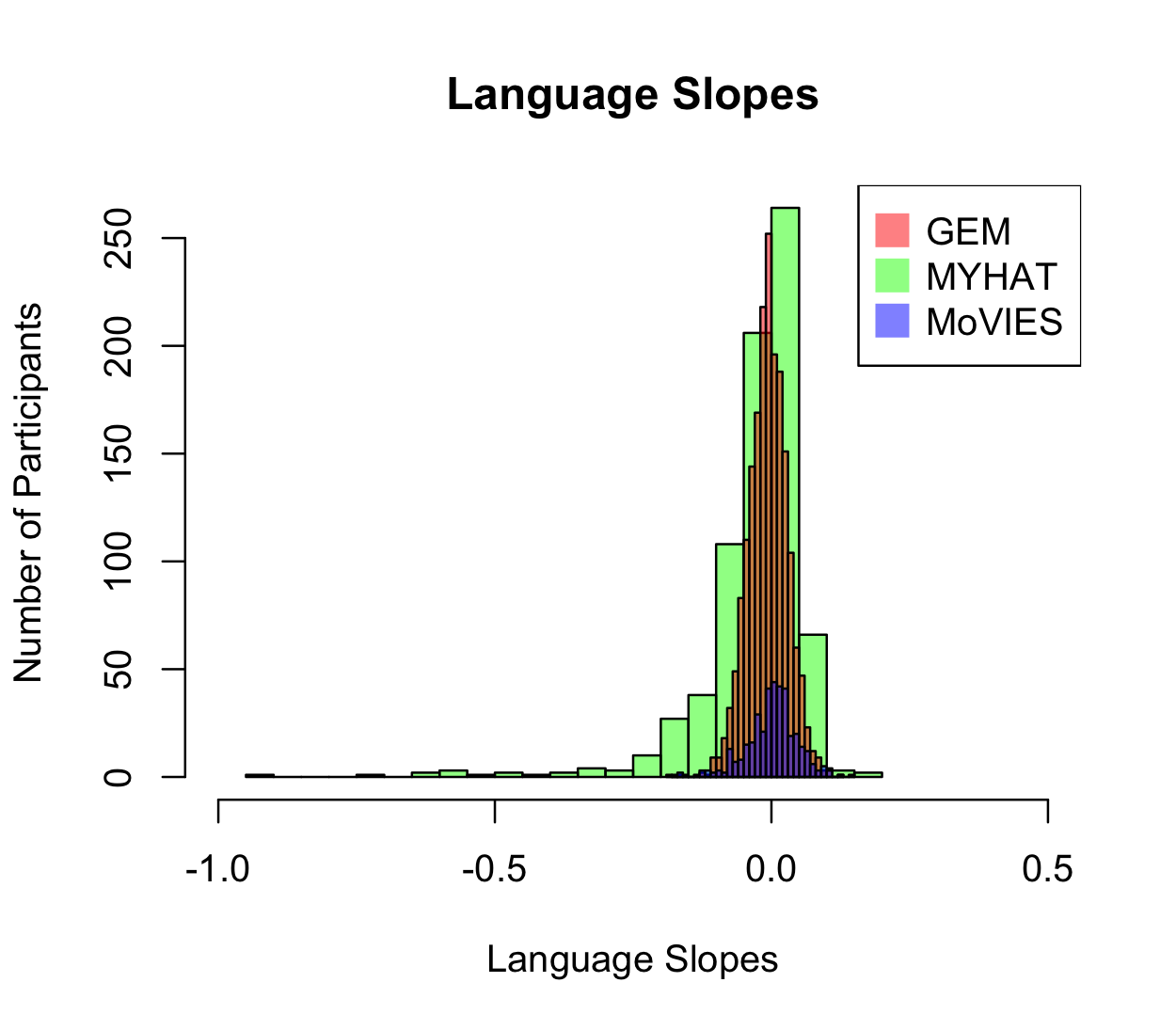

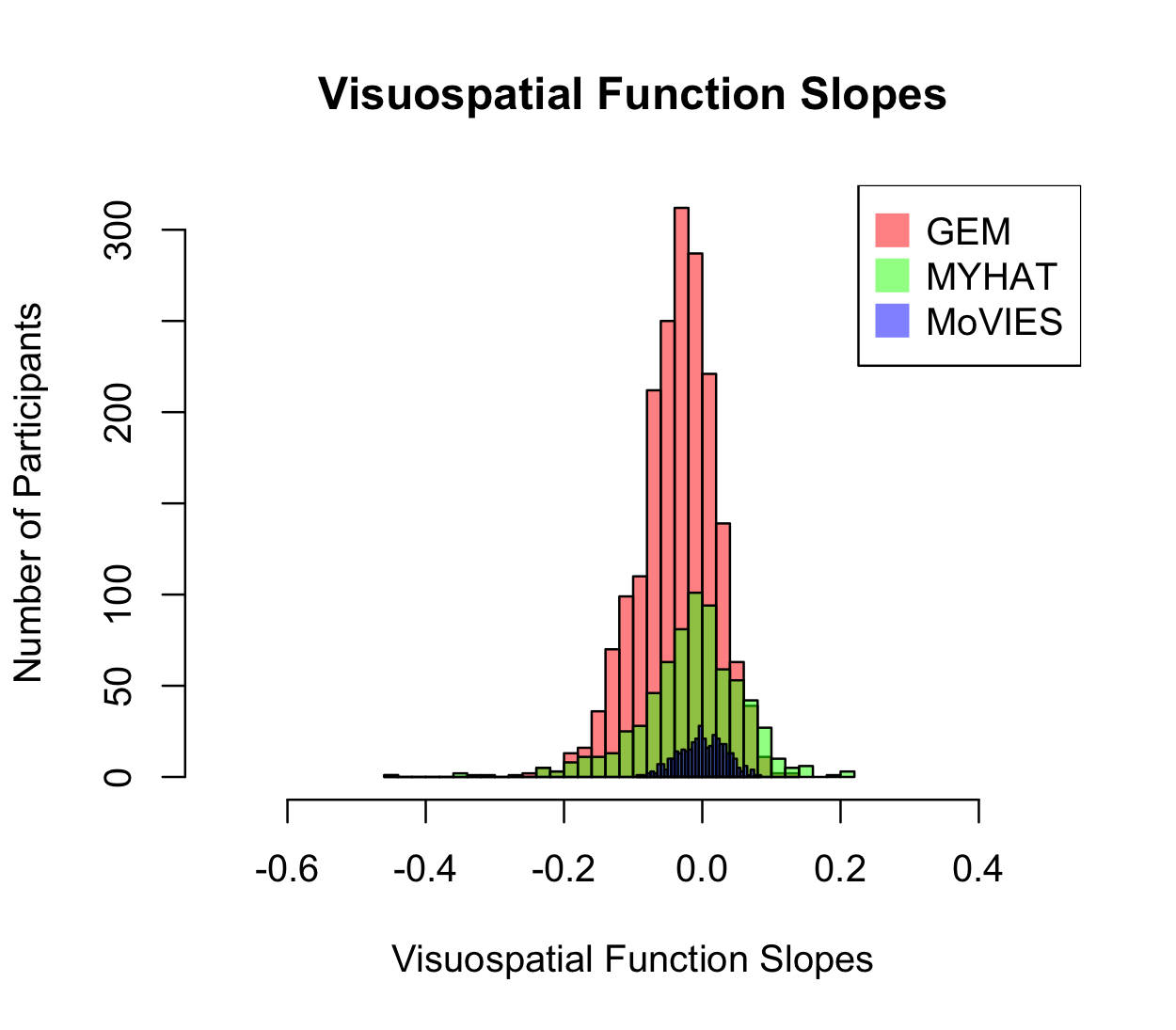

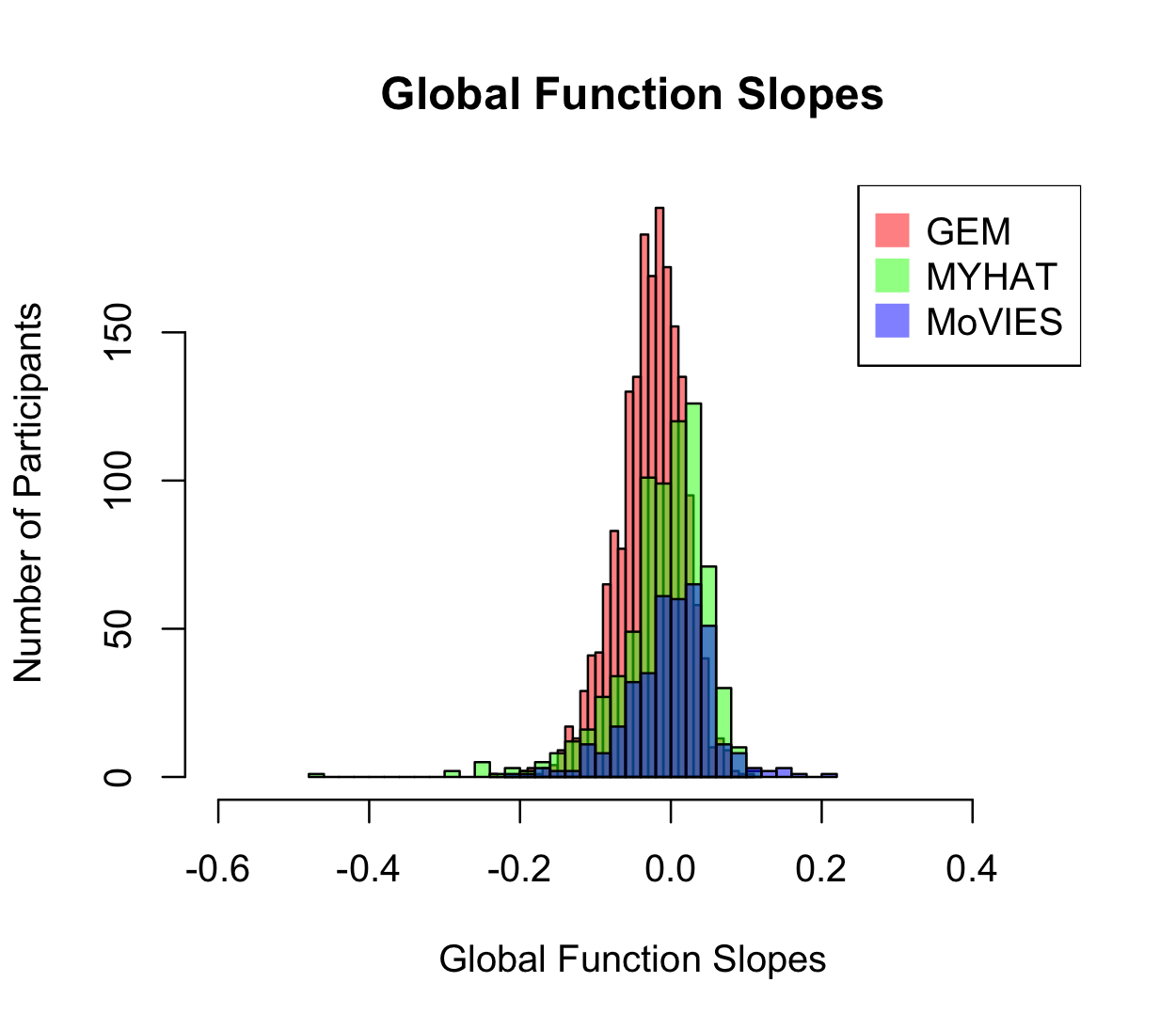


**Figure S1: Distribution of cognitive slopes across the three longitudinal cohorts : Gingko Evaluation of Memory (GEM), Monongahela-Youghiogheny Healthy Aging Team (MYHAT), and the Monongahela Valley Independent Elders Survey (MoVIES).**


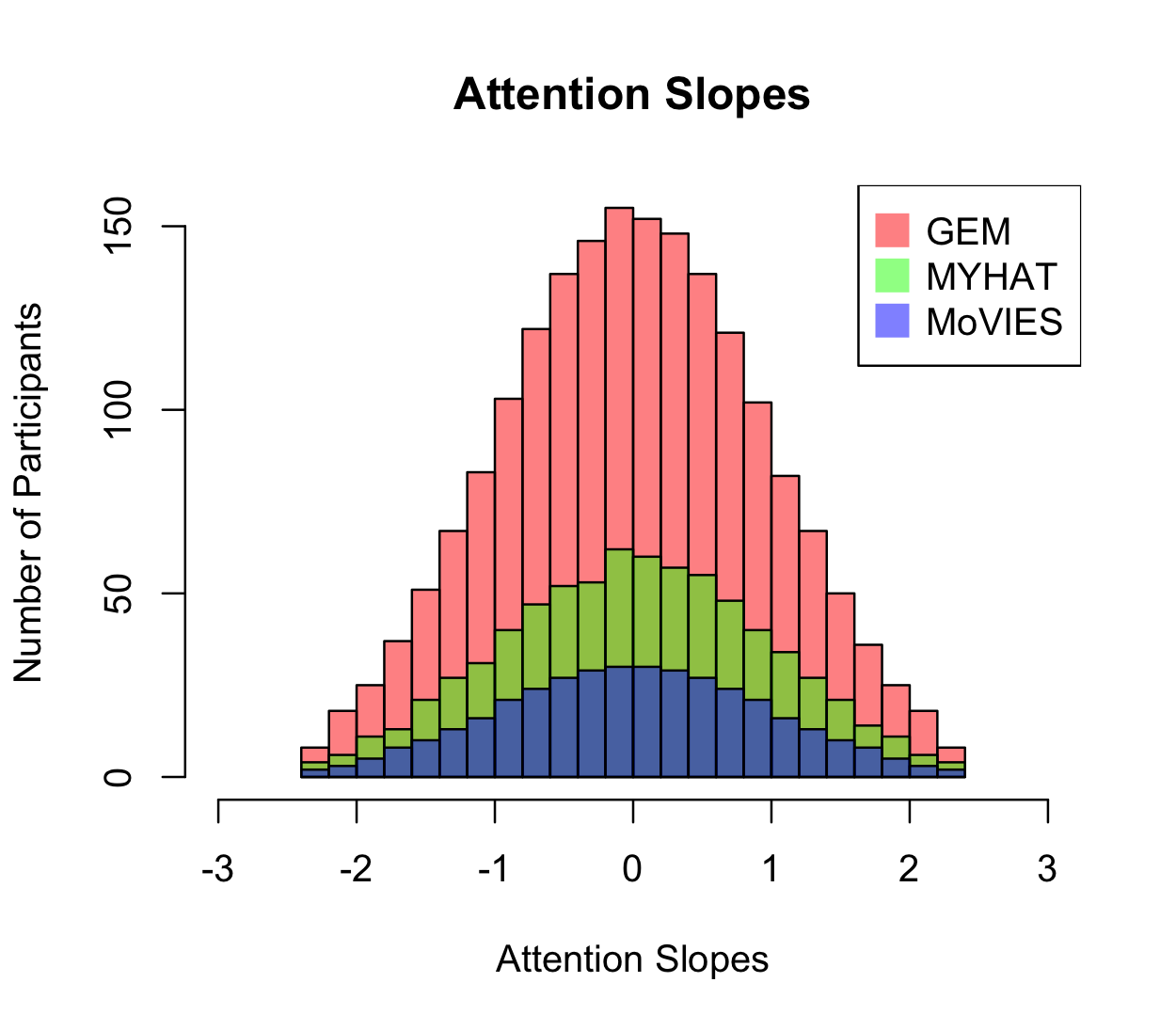

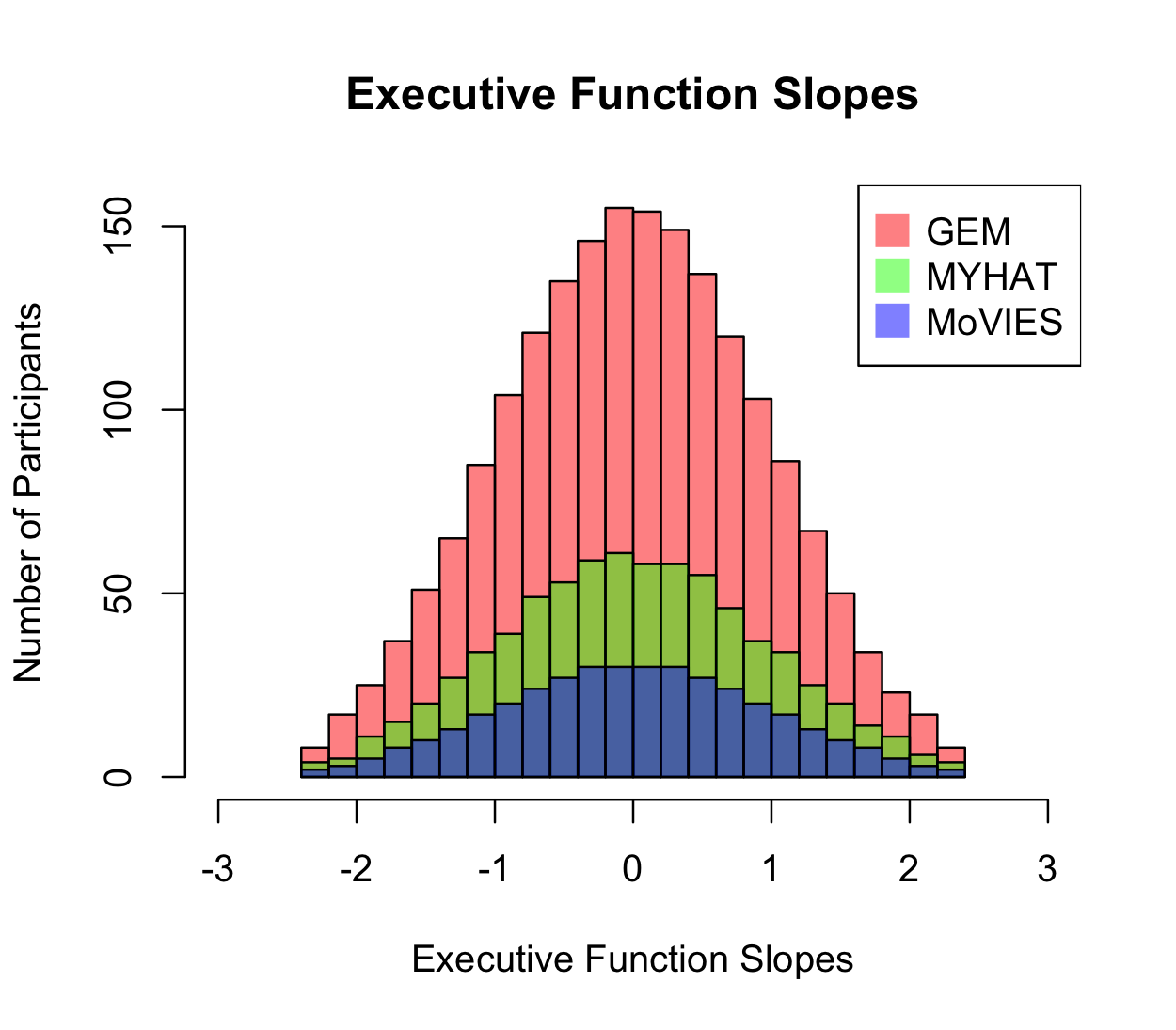

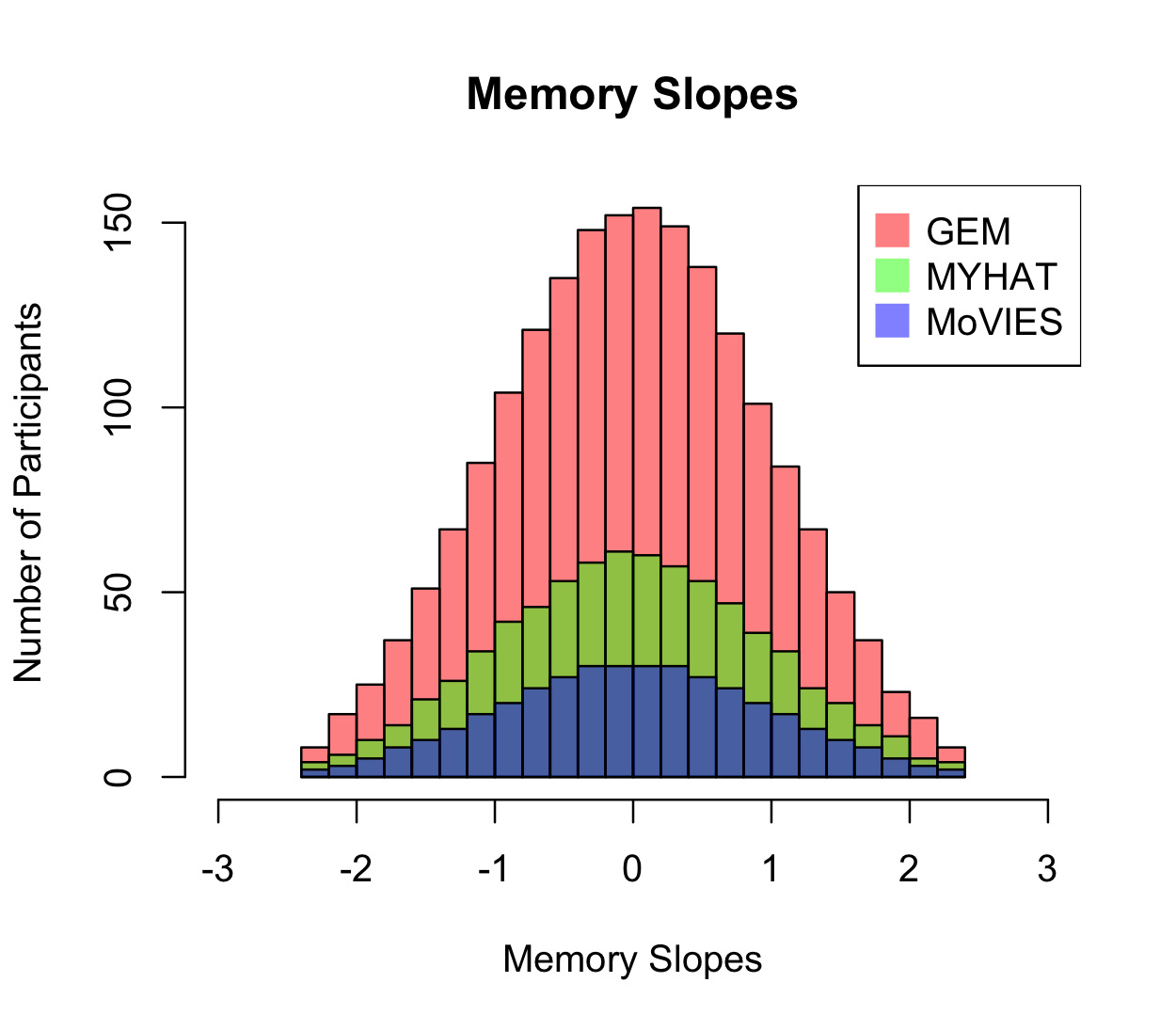

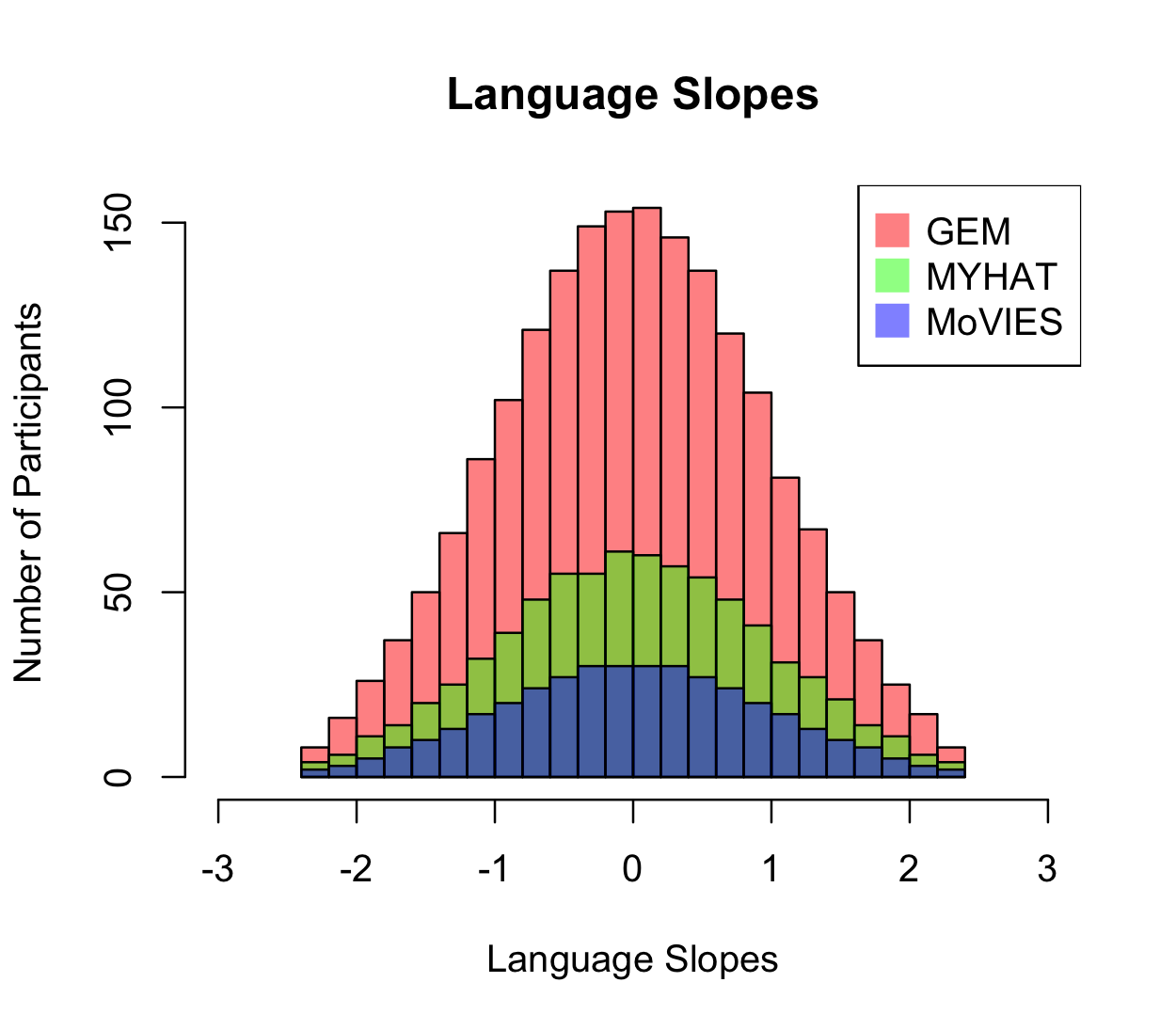

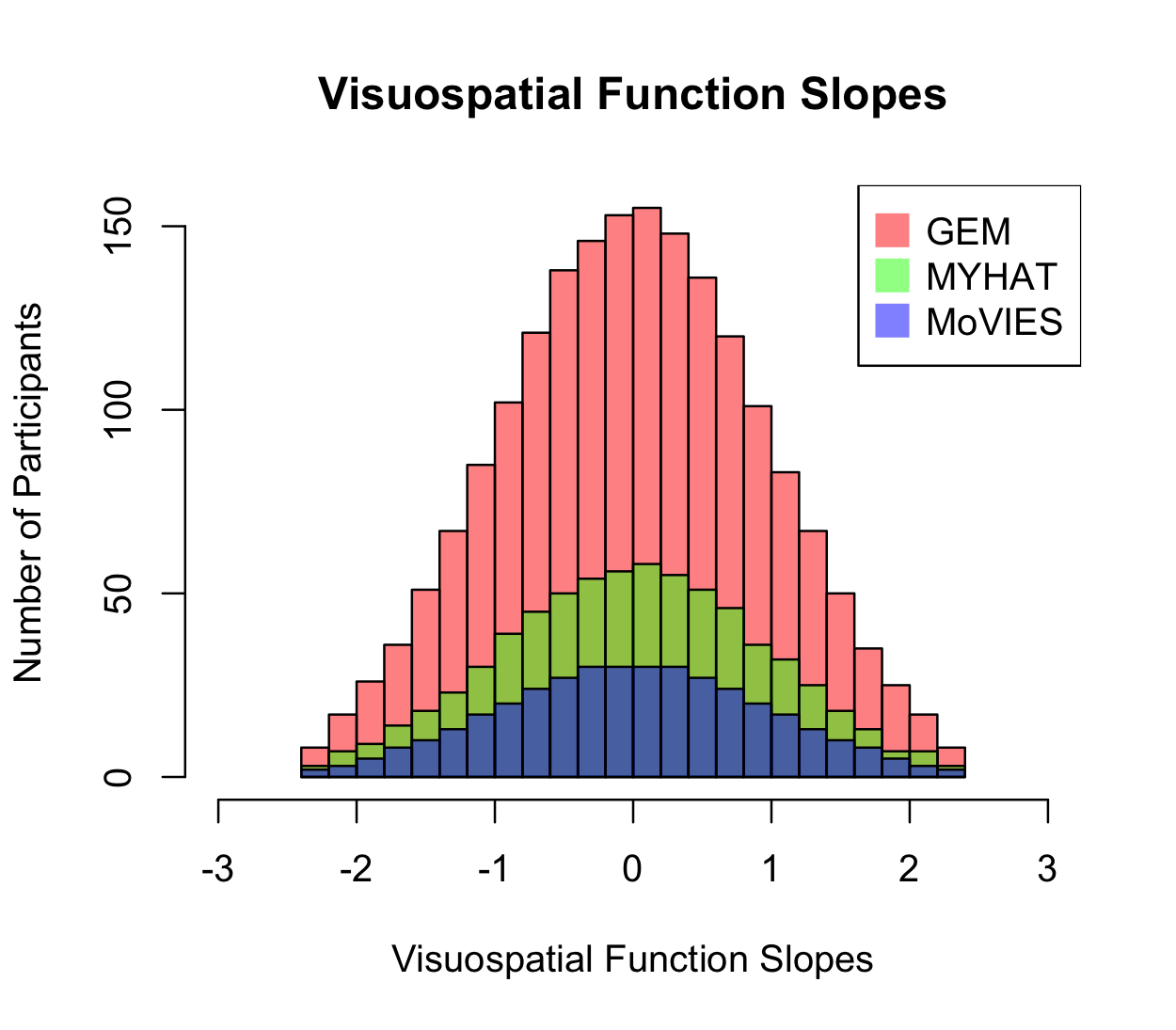

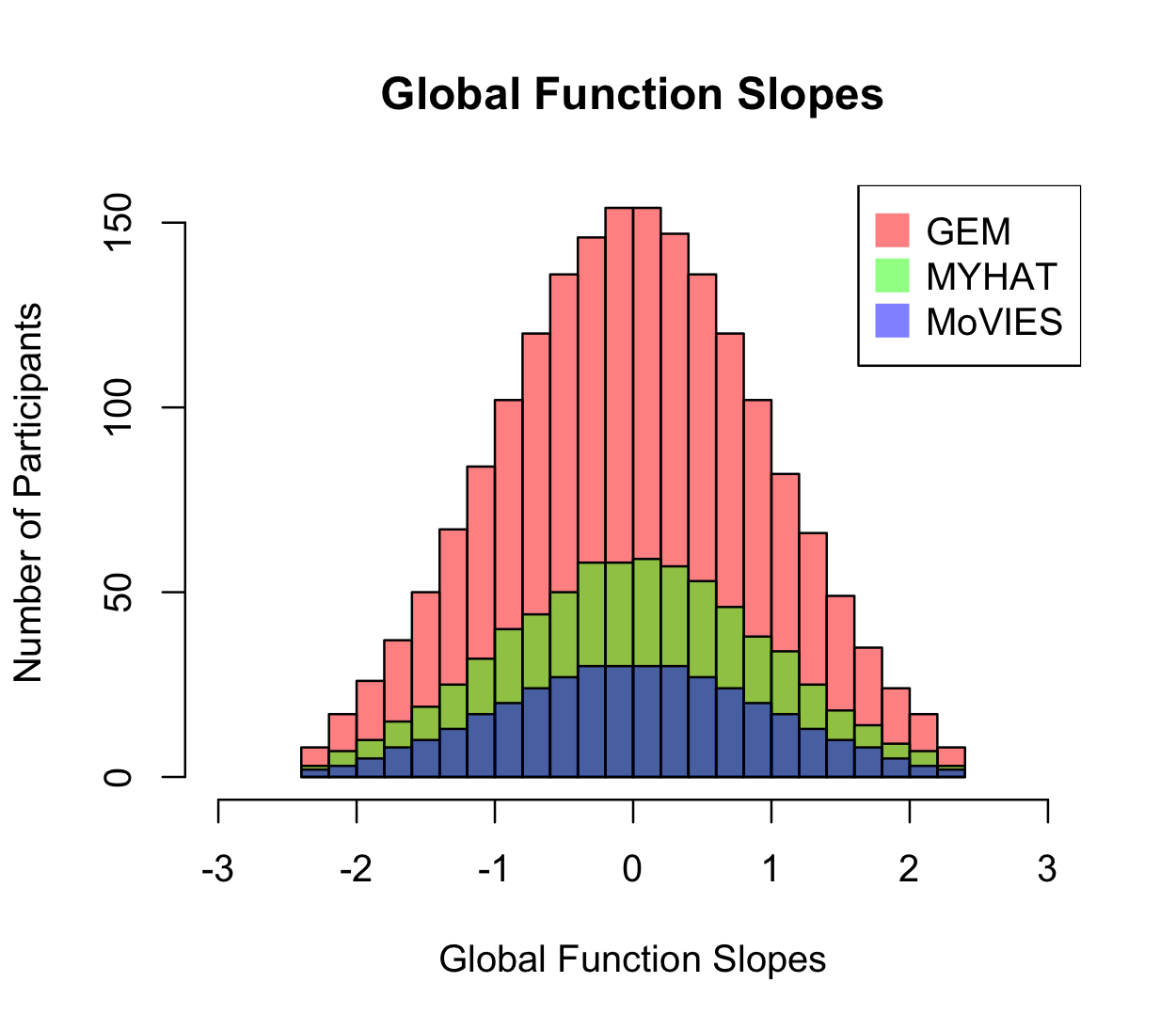


**Figure S2: Distribution of normalized and transformed cognitive slopes across the three longitudinal cohorts, Gingko Evaluation of Memory (GEM), the Monongahela-Youghiogheny Healthy Aging Team (MYHAT)****, and the Monongahela Valley Independent Elders Survey (MoVIES).**


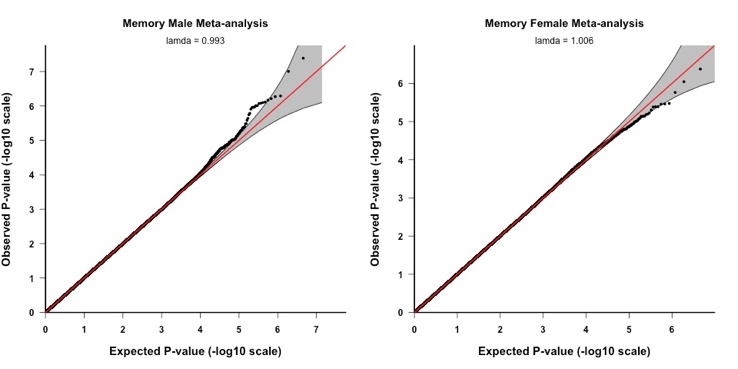

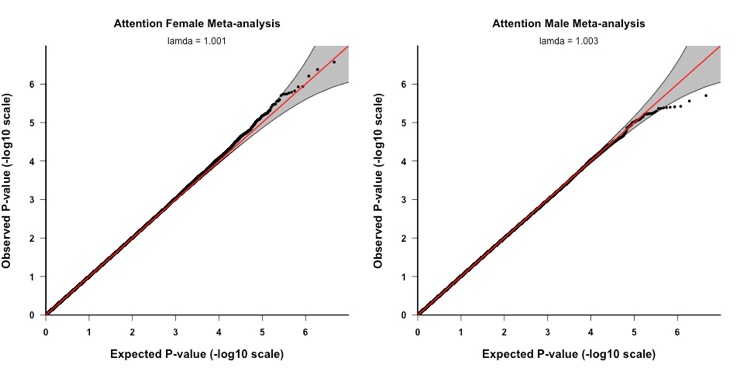


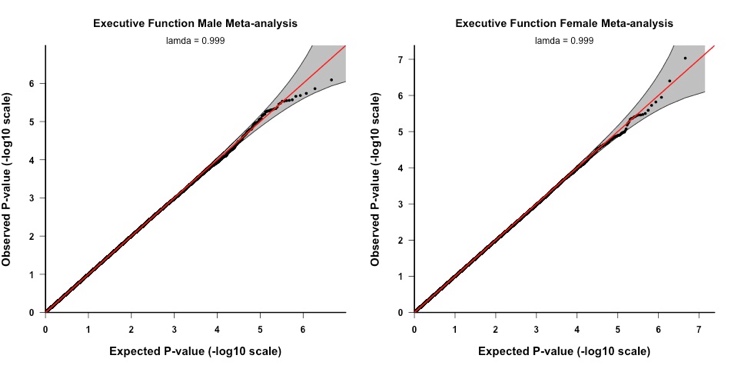

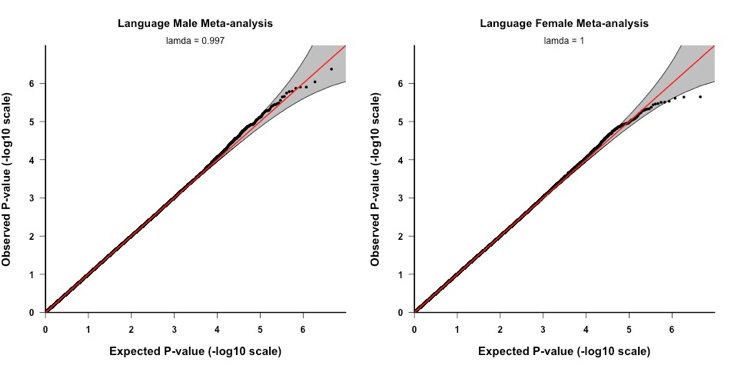


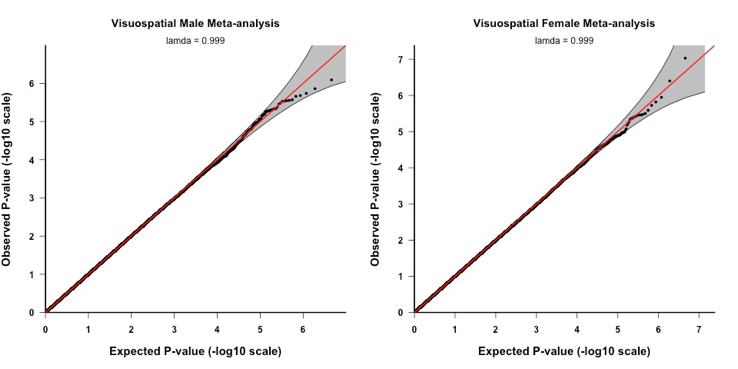

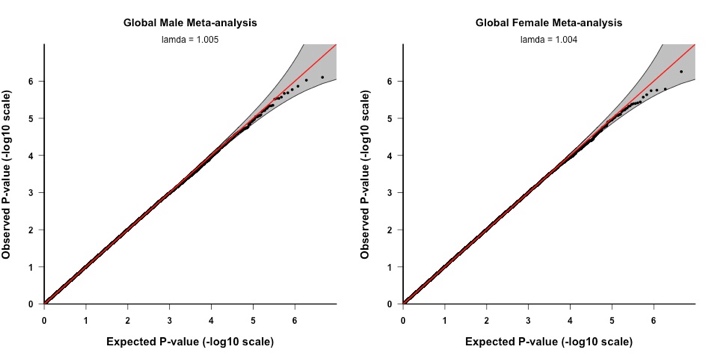


**Figure S3: QQ plots and the genomic inflation factor (λ) values of the meta-analysis of decline of memory, attention executive function, language, visuospatial function and global function in males and females.**


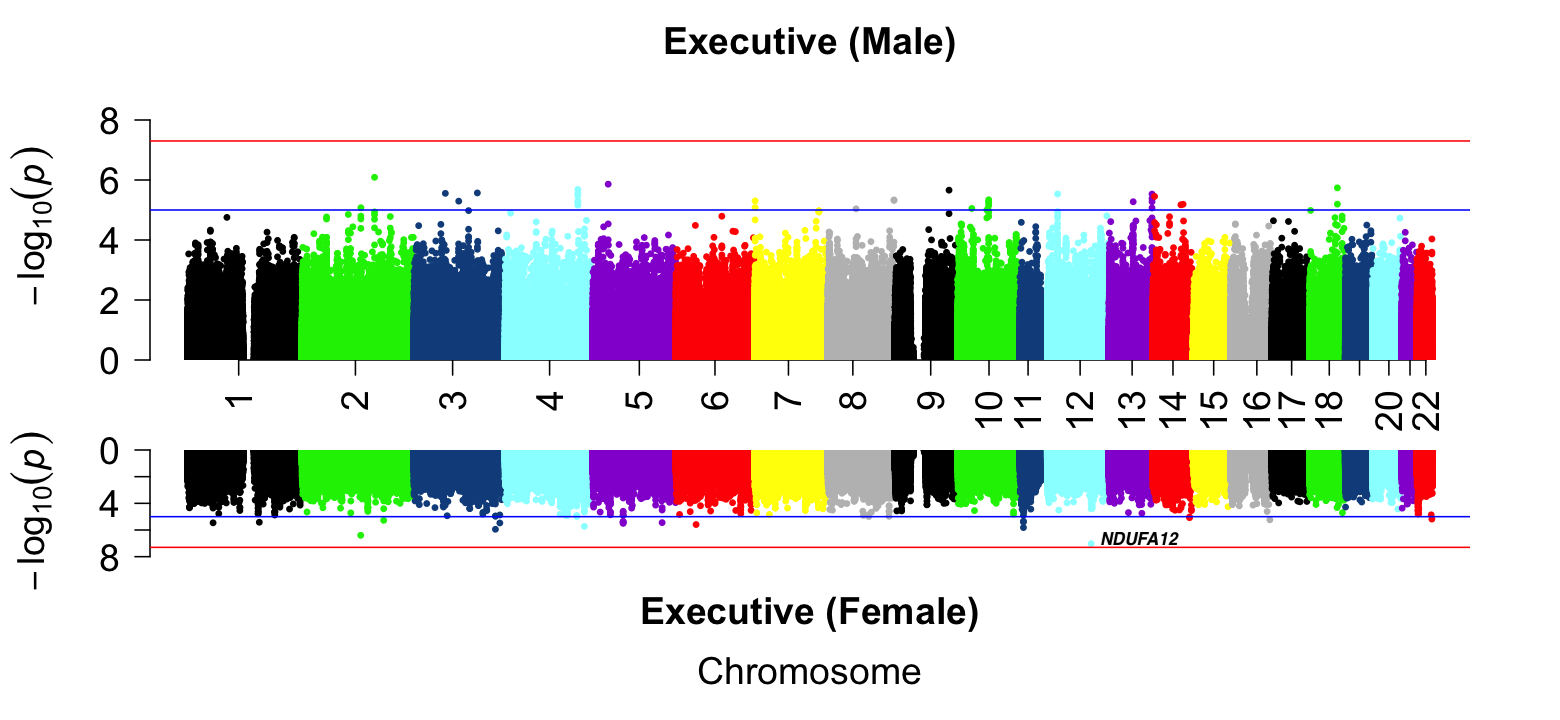


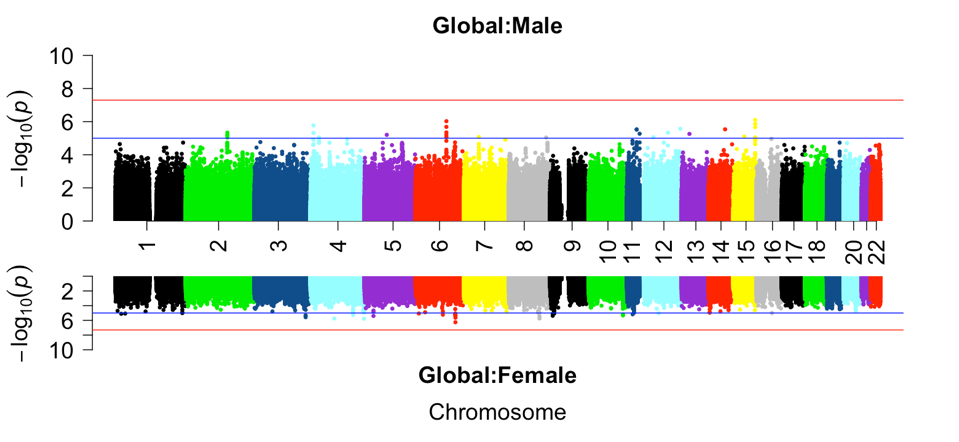


**Figure S4: Miami Plots of the meta-analysis of decline of executive function (top), global cognitive function (bottom) in males and females.**


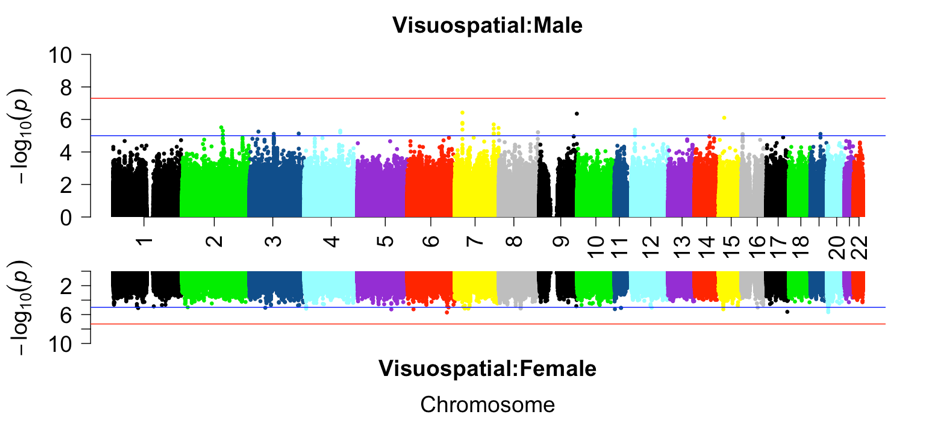


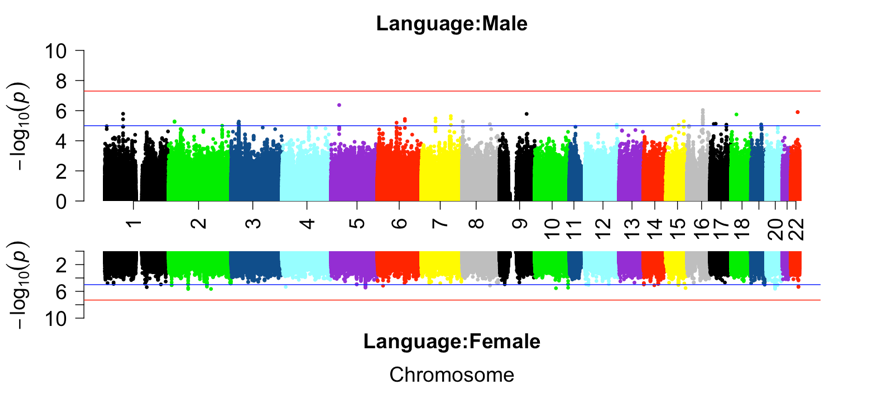


**Figure S5: Miami Plots of the meta-analysis of decline of visuospatial function(top) and language (bottom) in males and females.**


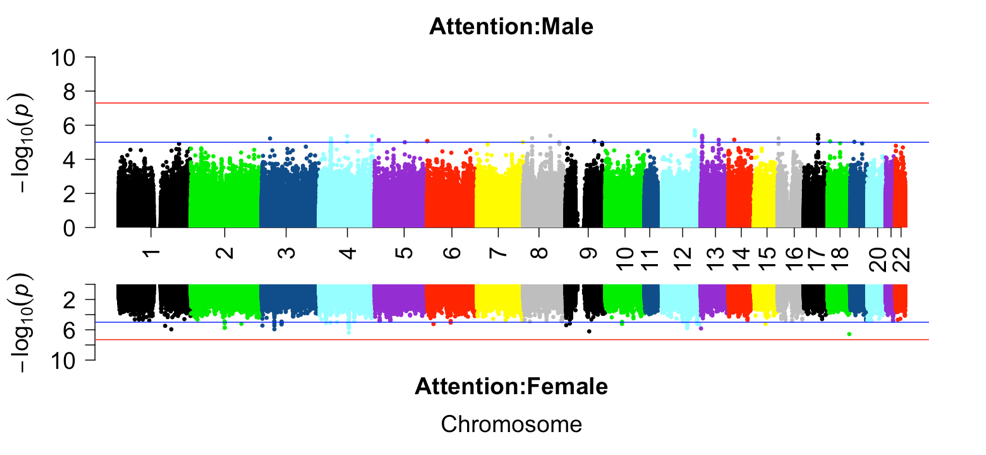


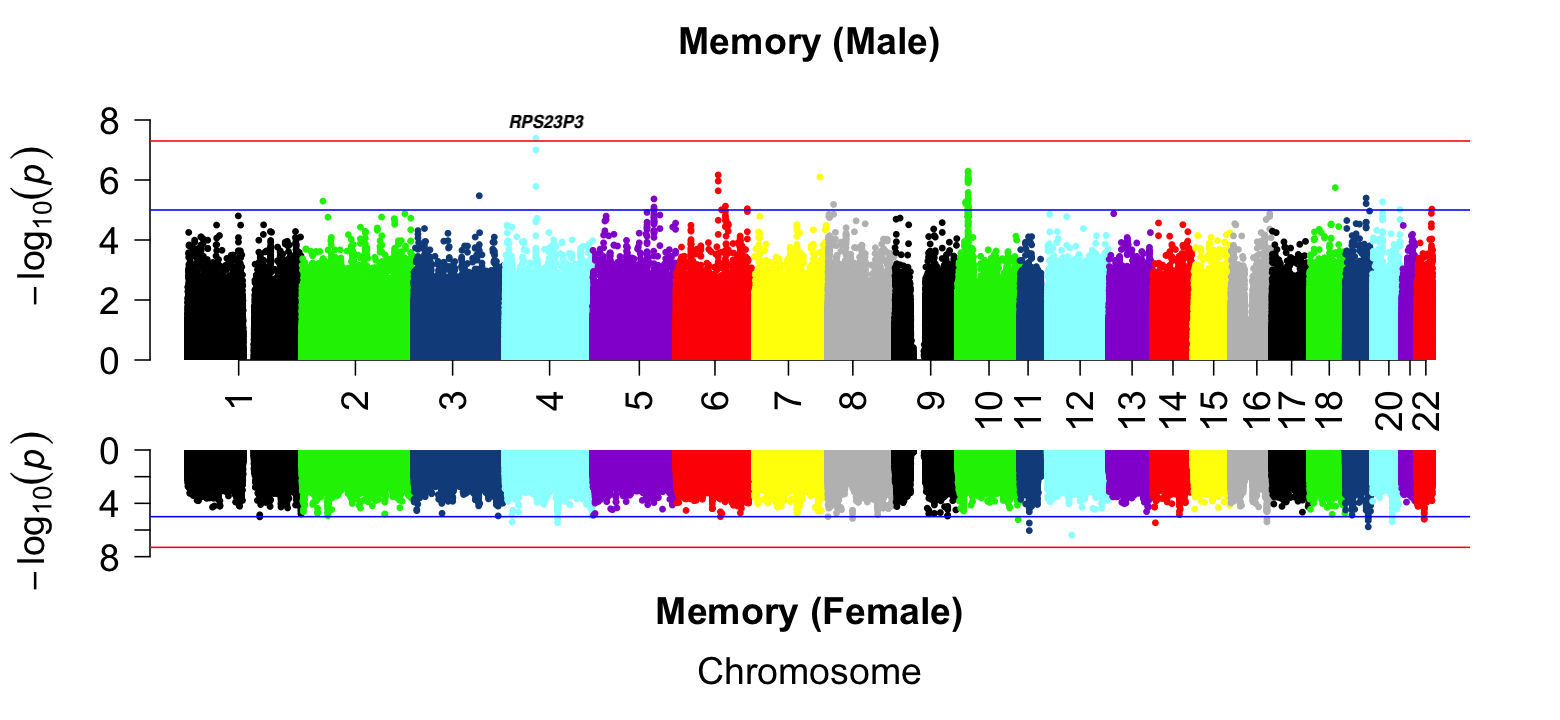


**Figure S6: Miami Plots of the meta-analysis of decline of attention (top) and memory (bottom) in males and females.**
